# Long-chain glucomannan supplementation modulates immune responsiveness, as well as intestinal microbiota, and impacts infection of broiler chickens with *Salmonella enterica* serotype Enteritidis

**DOI:** 10.1186/s13567-022-01026-z

**Published:** 2022-02-04

**Authors:** Nathalie Meijerink, Jean E. de Oliveira, Daphne A. van Haarlem, David M. Lamot, Francisca C. Velkers, Hauke Smidt, J. Arjan Stegeman, Victor P. M. G. Rutten, Christine A. Jansen

**Affiliations:** 1 Department of Biomolecular Health Sciences, Division of Infectious Diseases and Immunology, Faculty of Veterinary Medicine, Utrecht University, Utrecht, The Netherlands; 2 Cargill R&D Center Europe, Vilvoorde, Belgium; 3 Cargill Animal Nutrition and Health Innovation Center, Velddriel, The Netherlands; 4 Department of Population Health Sciences, Division of Farm Animal Health, Faculty of Veterinary Medicine, Utrecht University, Utrecht, The Netherlands; 5 Laboratory of Microbiology, Wageningen University & Research, Wageningen, The Netherlands; 6 Department of Veterinary Tropical Diseases, Faculty of Veterinary Science, University of Pretoria, Pretoria, South Africa; 7 Present Address: Department of Animal Sciences, Cell Biology and Immunology Group, Wageningen University & Research, Wageningen, The Netherlands

**Keywords:** Long-chain glucomannan, *Salmonella* Enteritidis, immunity, NK cells, T cells, IELs, intestinal microbiota, poultry, broiler chickens

## Abstract

The zoonotic pathogen *Salmonella enterica* serotype Enteritidis (SE) causes severe disease in young chickens. Restriction on antibiotic use requires alternative SE control strategies such as nutritional solutions to improve the resistance of chickens. In this study, chickens were fed long-chain glucomannan (GM) or standard diet and challenged with SE at seven days of age. During 21 days post-infection (dpi), we determined numbers and responsiveness of natural killer (NK) and T cells in ileum and spleen, and SE-specific antibody titers in serum. Microbiota compositions in ileum and caeca were determined, as well as correlations of these with numbers and function of immune cells. Some of the samples in the control group had numerically higher CFUs than the GM-treated group. In addition, the relative abundance of SE based on DNA assessment was significantly lower at 21 dpi upon GM supplementation. At 3 dpi, numbers of intraepithelial NK cells were significantly higher, while activation of intraepithelial NK cells (7 dpi), numbers of intraepithelial cytotoxic CD8^+^ T cells (14 dpi) and SE-specific antibodies (14 dpi) were numerically higher. Furthermore, relative abundance of the commensal lactic acid bacteria (LAB) significantly increased with GM supplementation post-infection. Higher relative abundance of streptococci was associated with reduced SE in ileal and caecal contents at 21 dpi. Relative abundance of streptococci negatively correlated with SE counts and positively correlated with NK cell activation and SE-specific antibodies, which suggests involvement of the commensal LAB in NK cell responsiveness. These results indicate that GM supplementation modulates the immune system, intestinal microbiota and impacts SE infection of young chickens.

**Supplementary Information:**

The online version contains supplementary material available at 10.1186/s13567-022-01026-z.

## Introduction

*Salmonella enterica* serotype Enteritidis (SE) is a zoonotic pathogen that may cause severe disease and death in young chickens as well as subclinical infections in adult chickens [1]. Moreover, SE-contaminated poultry products are amongst the leading causes of foodborne diseases in humans [2]. Faecal salmonellae infect chickens via the oral or respiratory route, colonize the intestinal tract and disseminate to organs such as liver and spleen resulting in a systemic infection [3, 4]. Prevention of SE infection in poultry is thus important for health and welfare of chickens and to avoid substantial economic losses in the poultry sector and food recalls. In addition, SE prevention in poultry is relevant for the health and wellbeing of humans in terms of food safety as well as to avoid loss of productivity and medical costs [5, 6]. Therapeutic treatment of SE infection in chickens with antibiotics is restricted nowadays due to limited effectiveness against *Salmonella* strains, the risk of residues in poultry products, and potential induction of antibiotic resistance [7]. This encourages the search for immune-modulatory strategies to increase the resistance to SE.

Immune responsiveness in young chickens largely depends on maternal antibodies and the innate immune system, since the adaptive immune system is not fully developed yet [8, 9]. Natural killer (NK) cells are key players of innate immunity and are abundantly present among the intraepithelial lymphocytes (IELs) in the intestine, in addition to γδ T cells and cytotoxic CD8^+^ T cells [10–12]. Directly underneath the intestinal epithelium, macrophages, B cells and helper CD4^+^ T cells predominate [13, 14]. Apart from epithelial cells, IELs constitute the first cellular defense barrier in response to intestinal SE colonization. The important role of heterophils and macrophages in the early response to SE infection has been studied by others [15, 16]. In addition, we reported increased numbers of intraepithelial IL-2Rα^+^ and 20E5^+^ NK cells in the first days post SE infection. In parallel, NK cell activation was significantly enhanced in the IEL population and spleen of SE-infected compared to uninfected chickens, as reflected by increased CD107 and IFNγ expression as parameters for cytotoxicity and cytokine production respectively [17]. The adaptive immune system gradually takes over clearance of SE by responses of γδ T cells, cytotoxic T cells and antibody production [17–20]. Both γδ and cytotoxic T cells can express the CD8αα or CD8αβ co-receptor. The role of CD8αα^+^ T cells has not been fully elucidated in contrast to the antigen-experienced CD8αβ^+^ cytotoxic responses. Studies in humans and mice showed that CD8αα^+^ T cells are involved in maintaining the integrity of the intestinal barrier as well as in NK-like cytotoxicity in response to pathogens [21–23]. In a previous study we observed increased numbers of both intraepithelial CD8αα^+^ and CD8αβ^+^ γδ T- and cytotoxic T cells in response to SE infection, which were more pronounced for the CD8αα^+^ subset suggesting an important role for these T cells in defense against pathogens [17]. We hypothesize that stimulation of intraepithelial NK and T cells through feed supplementation during the early life of chickens may aid in increasing their resistance to SE infection.

Nutritional solutions have resulted in a reduction in colonization and shedding of salmonellae in chickens. Depending on the type of modulatory compounds, this was shown to occur directly through binding of feed additives to the bacteria, induction of competitive exclusion, modulation of the intestinal microbiota composition or by direct stimulation of numbers and function of immune cells [24–29]. In addition, changes in the intestinal microbiota by feed supplementation indirectly modulate the immune system, since interactions occur between commensal species and immune cells [30–33]. Feed additives that have shown to reduce *Salmonella enterica* strains when provided directly after hatch include probiotics [34–39], prebiotics [40–42] and polysaccharides [43]. Furthermore, administration of probiotics to embryos by in ovo inoculation has shown to reduce SE infection incidence after hatch [44, 45]. In a previous study we showed that long-chain glucomannan (GM), a water-soluble polysaccharide that acts as a prebiotic, increased numbers and activation of NK cells in the IEL population as well as the relative abundance of lactic acid bacteria (LAB) in the intestine [31]. However, it is not known yet whether and how the increased numbers and function of NK cells and the effects on microbiota composition upon GM supplementation increase the resistance to infections such as SE.

In this study, we investigated the protective potential and underlying mechanisms of GM supplementation, directly after hatch, against experimental SE challenge in seven day old broiler chickens. We analyzed effects of GM on the presence of SE in the intestine and spleen, numbers and functional aspects of NK and T cells in the IELs and spleen, serum antibody titers and intestinal microbiota composition until three weeks after challenge. The findings of the present study contribute to understanding the effects of feed supplementation and show modulation of the immune system, changes in microbiota and some impact on SE infection. Strategies to improve immune-mediated resistance of young broiler chickens to infections may contribute to reducing zoonotic infections as well as use of antibiotics, hence increasing both animal health and welfare, and food safety for humans.

## Materials and methods

### Animals and husbandry

A total of 72 Ross 308, 17- and 18-day old embryonated eggs were obtained from the same parent flock of a commercial hatchery (Lagerwey, The Netherlands). The parent flock tested negative for *Salmonella* and was treated with AviPro Salmonella DUO according to standard procedures of the commercial hatchery. Upon arrival at the facilities of the Department of Population Health Sciences, Faculty of Veterinary Medicine, Utrecht University, The Netherlands, eggs were disinfected with 3% hydrogen peroxide, a standard procedure in the facilities [30] and placed in disinfected egg hatchers in one stable. Directly upon hatch, chickens were weighed, labelled and randomly housed according to their feed group in two floor pens separated by a solid wall. Both female and male chickens were included in the study, equally distributed across feed groups and none of the chickens received vaccination. Each pen was divided in two equal subunits (A, B). Pens were lined with wood shavings (2 kg/m^2^), and water and feed was provided ad libitum. Standard (control, *n*  = 36) or long-chain glucomannan supplemented [GM; 0.2% GM inclusion in complete standard diet (100%), *n*  = 36] *Salmonella*-free commercial starter and grower feeds were provided (Research Diet Services, The Netherlands). A standard lighting and temperature scheme for Ross broiler chickens was used, equal for both pens.

The animal experiment was approved by the Dutch Central Authority for Scientific Procedures on Animals and the Animal Experiments Committee (Registration Number AVD1080020174425) of Utrecht University (The Netherlands) and all procedures were done in full compliance with all relevant legislation.

### Experimental design

To determine microbiota composition of the environment, swabs (FLOQSwabs^®^, COPAN, Italy) of the hatchers and floor pens were taken before hatching and once more of the hatchers after hatching. Swabs were stored at room temperature (RT) in 0.5 mL DNA/RNA Shield (Zymo Research, CA, USA) until DNA purification. Additional swab samples of the hatchers after hatching were taken to confirm that embryos were *Salmonella*-free by analyzing bacterial counts on MSRV plates (Veterinair Centrum Someren, The Netherlands). At three days post-hatch, six chickens per feed group (three per subunit A/B) were randomly selected and sacrificed, for collection of ileum (± 10 cm distal from Meckel’s diverticulum) and spleen to confirm absence of SE before experimental inoculation. Before inoculation, at seven days post-hatch [0 days post-infection (dpi)], six chickens per feed group (three per subunit A/B) were randomly selected and sacrificed to collect ileum, spleen and contents of ileum (distal from Meckel’s diverticulum) and caeca to determine the baseline levels of the various parameters and once more absence of SE. Also, intestinal contents were collected using a sterile plastic cell culture loop, subsequently transferred into 2 mL sterile tubes containing 0.5 mL DNA/RNA Shield (Zymo Research), and stored at RT for DNA extraction. Chickens of both groups were challenged at 0 dpi by oral inoculation with 0.25 mL brain heart infusion (BHI) medium containing 1.55 × 10^6^ SE colony-forming units (CFUs) in the control group and 1.78 × 10^6^ SE-CFUs in the GM group. At 3, 7, 14 and 21 dpi, six chickens per group (three per subunit A/B) were randomly selected, weighed prior to post-mortem analyses to calculate body weight gain and sacrificed for collection of ileum, spleen and contents of ileum and caeca to determine bacterial CFUs, numbers and function of NK cells and T cells, and microbiota composition. In addition, at 0, 7, 14 and 21 dpi, blood (at least 5 mL) was collected in EDTA tubes (VACUETTE^®^ K3E EDTA, Greiner Bio-One, The Netherlands) for determination of SE-specific antibody levels. The use of six chickens per group per time point was calculated using power analysis (Sample size and power calculator, LASEC, China). To calculate absolute cell numbers, ileum segments and spleens were weighed immediately after collection of the tissues, prior to isolation of cells.

### SE culture

*Salmonella enterica* serotype Enteritidis (strain K285/93 Nal^res^) was kindly provided by Dr. E. Broens, Veterinary Microbiological Diagnostic Center, the Faculty of Veterinary Medicine, Utrecht University, and cultured as described previously [46]. In short, from an overnight culture of the SE strain on blood agar (Oxoid, The Netherlands) two single colonies were used to inoculate two volumes of 45 mL BHI medium (Oxoid), and both cultures were incubated aerobically overnight and 37 °C in a shaking (200 rpm) incubator (Certomat BS-1, B. Braun Biotech international, Sweden). Samples of the SE cultures were diluted 1:10 in PBS were and OD values measured using a Ultrospec 2000 (Pharmacia Biotech, Sweden). SE concentrations were calculated from a previously determined growth curve, and SE were diluted in BHI medium to 4 × 10^6^ CFUs/mL, to constitute the inocula. The SE challenge dose was based on previous studies [17, 47]. Serial dilutions of the two inocula were plated for overnight culture and number of CFUs were counted to determine the exact SE concentrations; 6.20 × 10^6^ CFUs/mL (control) and 7.12 × 10^6^ CFUs/mL (GM).

### Quantitation of SE in ileum and spleen

At −4, 0, 7, 14 and 21 dpi, the numbers of *Salmonella* colonies on the plates were counted to assess numbers of SE in ileum and spleen. From the cell suspensions of either the ileum segments or homogenized spleens, 100 μL was plated with a spatula on RAPID’ *Salmonella* Medium plates (Bio-Rad, The Netherlands). Plates were incubated overnight at 37 °C and the purple colonies were counted. SE numbers were expressed as CFU per gram tissue to depict SE load as well as number of chickens positive for SE, as has been described previously [46]. The limit of detection (LOD) was 100 CFU per gram tissue.

### Isolation of cells and serum

Isolation of IELs from ileum and leukocytes from spleen was conducted according to the procedure described previously [12, 48]. Briefly, ileum segments were washed with PBS to remove contents, cut in sections of 1 cm^2^ and washed again. Subsequently, the IELs were collected by incubating the sections three times for 15 min in a shaking incubator (Certomat BS-1) at 200 rpm and 37 °C in 20 mL EDTA-medium: HBSS 1 × (Gibco^®^) supplemented with 10% heat-inactivated FCS (Lonza) and 1% 0.5 M EDTA (UltraPure™, Invitrogen, The Netherlands). Supernatants were collected and centrifuged for 5 min at 335 × *g* and 20 °C (Allegra™ X-12R Centrifuge, Beckman Coulter, The Netherlands). Pellets were then resuspended in PBS at a concentration of 10 mL per gram tissue and an aliquot of 100 µL was used for quantitation of SE. PBS was added to the remaining suspension up to 20 mL and IELs were isolated using Ficoll-Paque Plus (GE Healthcare, The Netherlands) density gradient centrifugation for 12 min at 673 × *g* and 20 °C, washed in PBS by centrifugation for 5 min at 393 × *g* and 4 °C and resuspended at 4.0 × 10^6^ cells/mL in complete medium (IMDM 2 mM glutamax I supplemented with 8% heat-inactivated FCS (Lonza), 2% heat-inactivated chicken serum, 100 U/mL penicillin and 100 µg/mL streptomycin; Gibco^®^). Spleens were homogenized using a 70 µm cell strainer (Beckton Dickinson (BD) Biosciences, NJ, USA) and the single-cell suspension was diluted in PBS at a concentration of 10 mL per gram tissue. An aliquot of 100 µL was used again for SE quantitation. Next, leukocytes were isolated by Ficoll-Paque Plus density gradient centrifugation (20 min, 1126 × *g*, 20 °C), washed in PBS and resuspended at 4.0 × 10^6^ cells/mL in complete medium as described for ileum.

**Figure 1:**
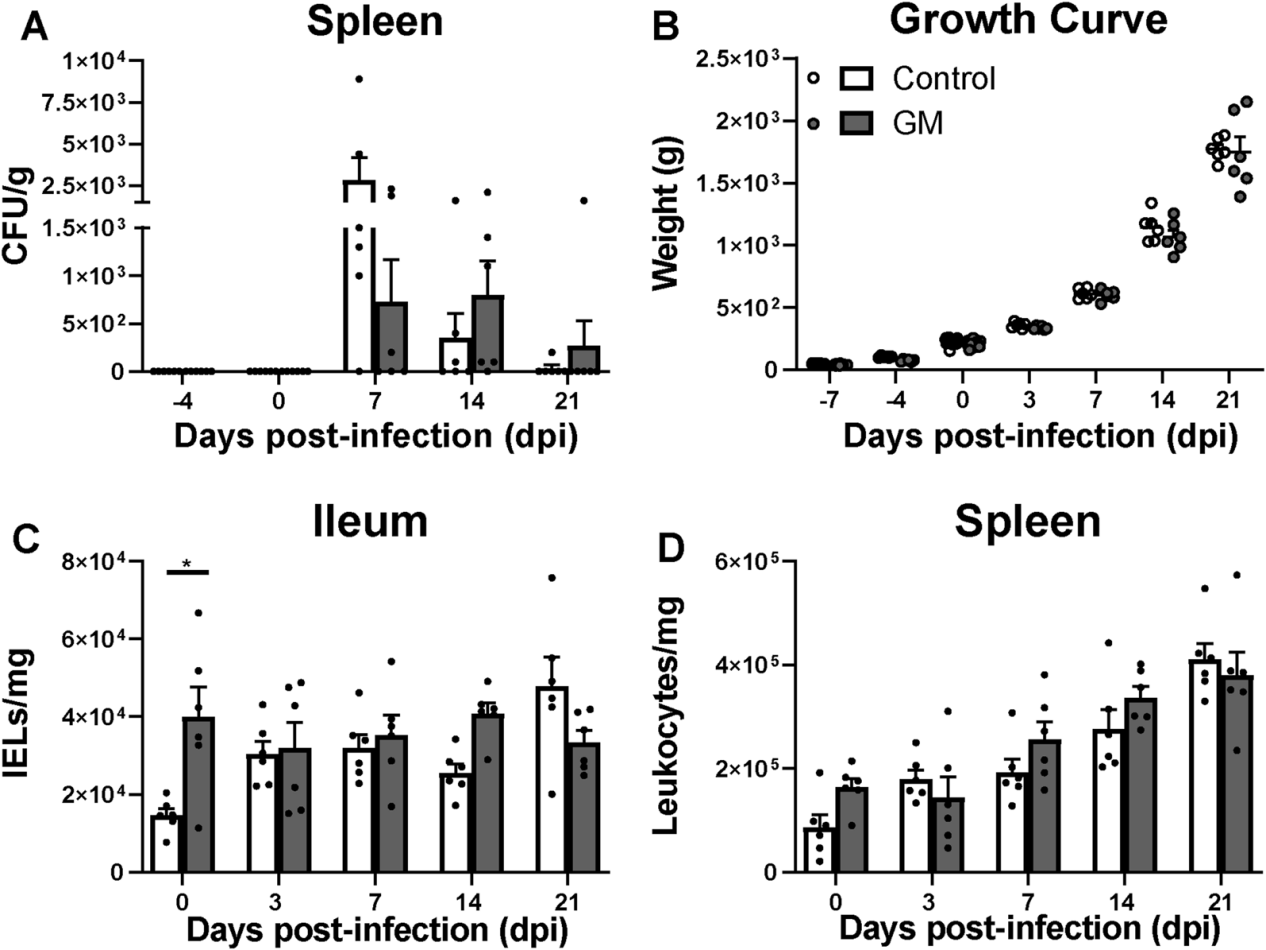
**Effect of GM on SE load, chicken growth and numbers of IELs and splenic leukocytes before and during infection in broiler chickens. A** *Salmonella enterica* serotype Enteritidis (SE, CFU/g) in the spleen of chickens either fed standard (control) or long-chain glucomannan supplemented (GM) diet before and during SE infection. **B** Body weights (g) of chickens either fed standard or GM diet in course of time before and during SE infection. **C** Numbers (cells/mg) of IELs in the ileum and **D** leukocytes in the spleen of chickens fed standard or GM diet in course of time before and during SE infection. Mean  +  SEM per diet group and time point are shown (*n*  = 6) and statistical significance between diet groups is indicated as *(*p*  < 0.05).

After isolation of IELs from ileum and leukocytes from spleen, cell numbers in the resulting suspensions were calculated using disposable counting chambers according to the manufacturer’s instructions (Glasstic Slide 10 with Grids, Kova International, CA, USA). The total cell numbers were used to calculate IELs per mg ileum or leukocytes per mg spleen (Figures 1C, D). To calculate the absolute numbers of NK and T cells within the live IEL or leukocyte populations, the percentages of cells positive for the markers expressed on these cell types, as determined in flow cytometry analyses, were used (Additional file 1, [17]). Absolute cell numbers were calculated using the following formula: (absolute number IELs or leukocytes per mg tissue)  ×  (percentage positive cells in the gate of interest of the live lymphocyte population).

Whole blood was allowed to coagulate for 1 h at RT, centrifuged for 10 min at 2095 × *g* and 15 °C, subsequently serum was collected and stored at −20 °C until further use.

### Phenotypic characterization of lymphocytes by flow cytometry

At 0, 3 and 7 dpi, numbers of NK and T cell subsets among IELs and splenocytes were measured. At 14 and 21 dpi only the number of T cell subsets was determined. Lymphocytes (1 × 10^6^) were stained with a panel of antibodies specific for surface markers known to be expressed on NK cells, as well as with anti-CD3 to enable exclusion of T cells from analyses. Another aliquot of cells was stained with a panel of antibodies specific for surface markers that distinguish γδ^+^, CD4^+^ and CD8^+^ T cell subsets (Table 1). Staining with primary and secondary antibodies (Table 1) was performed in 50 µL PBA (PBS (Lonza) containing 0.5% bovine serum albumin and 0.1% sodium azide). Cells were incubated for 20 min at 4 °C in the dark with staining antibodies and washed twice by centrifugation for 5 min at 393 × *g* and 4 °C either in PBA after incubation with the primary antibodies, or in PBS after incubation with the secondary antibodies. Subsequently, to be able to exclude dead cells from analysis, lymphocytes were stained in 100 µL PBS with a viability dye according to the manufacturer’s instructions (Zombie Aqua™ Fixable Viability Kit, Biolegend, The Netherlands) for 15 min at RT in the dark, washed twice in PBA and resuspended in 200 µL PBA. Of each sample, either 150 µL or a maximum of 1 × 10^6^ viable lymphocytes were measured using a CytoFLEX LX Flow Cytometer (Beckman Coulter), and data were analyzed with FlowJo software (FlowJo LCC, BD Biosciences). The gating strategies used to enable analyses of numbers and function of NK cells, γδ T cells and cytotoxic CD8^+^ T cells in the ileum are depicted in Additional file 1. The same gating strategy was used in the spleen and has been shown previously [17].

**Table 1:** **Flow cytometry staining reagents**.

Cell population	Primary antibody (mouse-anti-chicken)	Clone/isotype	Secondary antibody
NK cells	CD45-FITC^a^	LT40/IgM	–
	CD3-APC^a^	CT3/IgG1	–
	IL-2Rα-UNL^b^	28–4/IgG3	Goat-anti-mouse-IgG3-PE^a^
	20E5-BIOT^b^	IgG1	Streptavidin (SA)-PercP^e^
T cells	CD3-PE^a^	CT3/IgG1	–
	CD4-APC^a^	CT4/IgG1	–
	TCRγδ-FITC^a^	TCR-1/IgG1	–
	CD8α-UNL^a^	EP72/IgG2b	Goat-anti-mouse-IgG2b-APC/Cy7^a^
	CD8β-BIOT^a^	EP42/IgG2a	SA-PercP^e^
Assay			
CD107	CD107a-APC^c^	LEP-100 I 5G10/IgG1	–
	CD41/61-FITC^d^	11C3/IgG1	–
	CD3-PE^a^	CT3/IgG1	–
	CD8α-UNL^a^	EP72/IgG2b	Goat-anti-mouse-IgG2b-Alexa Fluor (AF) 790^f^
IFNγ	CD3-PE^a^	CT3/IgG1	–
	TCRγδ-FITC^a^	TCR-1/IgG1	–
	CD8α-UNL^a^	EP72/IgG2b	Goat-anti-mouse-IgG2b-AF790^f^
	IFNγ-APC^g^	MAb80/IgG1	–

Manufacturers: ^a^Southern Biotech, AL, USA; ^b^Purified antibody from hybridoma supernatant kindly provided by Göbel, T.W., Ludwig Maximilian University, Germany; ^c^Hybridoma obtained from Developmental Studies Hybridoma Bank (DSHB), University of Iowa, IA, USA; ^d^Serotec, United Kingdom; ^e^BD Biosciences; ^f^Jackson ImmunoResearch Laboratories, PA, USA; ^g^a kind gift from Lowenthal, J.W. CSIRO, Australia

### CD107 assay

At 0, 3, 7, 14 and 21 dpi, activation of NK cells and cytotoxic CD8^+^ T cells was determined in IELs and splenocytes. For this purpose the CD107 assay was used, which measures enhanced surface expression of CD107a that results from degranulation [48, 49]. Briefly, lymphocytes isolated from the IEL population and spleen were suspended in complete medium, and 1 × 10^6^ lymphocytes in 0.5 mL were incubated in the presence of 1 µL/mL GolgiStop (BD Biosciences) and 0.5 µL/mL mouse-anti-chicken-CD107a-APC for 4 h at 37 °C, 5% CO_2_. After incubation, lymphocytes were washed in PBA and stained as described above with monoclonal antibodies for NK and T cells, and in addition anti-CD41/61 to exclude thrombocytes from analyses, as mentioned in the CD107 panel (Table 1). Cells were washed in PBS, stained for viability and analyzed by flow cytometry.

### IFNγ assay

At 0, 3, 7, 14 and 21 dpi, expression of intracellular IFNγ was determined in NK cells, γδ T cells, CD8^+^ T cells and CD4^+^ T cells, using an assay adapted from Ariaans et al. [50]. Briefly, lymphocytes isolated from the IEL population and spleen were suspended in complete medium, and 1 × 10^6^ lymphocytes in 0.5 mL were incubated in the presence of 1 µL/mL Brefeldin A (Sigma Aldrich) for 4 h at 41 °C, 5% CO_2_. After incubation, lymphocytes were washed in PBA and stained as described above with surface markers summarized in the IFNγ panel (Table 1). Cells were washed in PBS, stained for viability and washed again in PBA. Next, lymphocytes were permeabilized differently as described by Ariaans et al. [50]. Here, lymphocytes were incubated in 200 µL of a mixture of BD FACS™ Permeabilizing Solution 2 and BD FACS™ Lysing Solution prepared according to manufacturer’s instructions (BD Biosciences) for 8 min at RT and immediately centrifuged for 2 min at 393 × *g* and 4 °C. Cells were washed twice in PBA, stained intracellularly with anti-IFNγ-APC in 50 µL PBA for 20 min at 4 °C in the dark, washed in PBA and finally analyzed by flow cytometry.

### SE-specific antibody titers in serum

To detect titers of SE-specific antibodies in the sera collected at 0, 7, 14 and 21 dpi, the commercially available *Salmonella* Enteritidis Antibody Test (IDEXX SE Ab X2 Test) was used according to manufacturer’s instructions (IDEXX Europe, The Netherlands). Positive and negative controls were included in the kit, and serum samples were analyzed in duplicate. Endpoint titers were calculated using the following formula:

10 ^ {1.5 ×  log_10_ [(sample µ − negative control µ)/(positive µ − negative control µ)]  +  3.47}.

### Microbiota composition of ileum and caeca

DNA was purified from ileal and caecal samples stored in DNA/RNA Shield using the ZymoBIOMICS DNA Kit according to manufacturer’s instructions (Zymo Research). Bacterial 16S ribosomal RNA genes were then amplified by running one PCR cycle while incorporating a cy-5 fluorescent labeled nucleotide, as described previously for labeling samples in microarray analysis [51]. Labeled PCR amplicons were then hybridized to a microarray chip coated with probes for intestinal bacteria previously selected as biomarkers for broiler performance and intestinal health [51]. Microarray annotation for probes included sequential numbers added after bacteria genus or species in order to avoid more than one probe with the same name. The microarray contains two probes for *Salmonella* and the *Salmonella* probe 2 has been validated to specifically capture *Salmonella enterica* serotype Enteritidis (Cargill Inc., proprietary). The fluorescence signal of each probe was read using a fluorescence array image reader (Sensovation AG, Germany). Fluorescence intensity of each probe was used as a parameter to determine relative abundance of each of the microbial taxa in the feed groups according to the experimental design.

In addition, Pearson’s correlations were calculated of immune cells and activation, SE-specific antibodies and SE-CFUs with intestinal microbial taxa that were significantly increased in each feed group before and during SE infection. Correlation (r) values from 0 to 1 (positive) and 0 to −1 (negative) are depicted in a heatmap, where 0–0.2 (0 to −0.2) is interpreted as no/negligible correlation, 0.2–0.5 (−0.2 to −0.5) as weak correlation, 0.5–0.8 (−0.5 to −0.8) as moderate correlation and 0.8–1 (−0.8 to −1) as strong correlation.

### Statistical analyses

First, the data were tested for normal distribution using the Shapiro–Wilk test. Differences between feed groups as well as within each group in the course of time in SE-CFUs per gram ileum and spleen, and SE-specific antibody titers in serum were analyzed using Kruskal–Wallis tests, followed by Dunn’s multiple comparisons tests. Differences between control and GM groups as well as within each group in the course of time in body weight, numbers of IELs or leukocytes, NK cell and T cell subsets as well as percentages of cells expressing CD107 and IFNγ in IELs and spleen were analyzed using one-way ANOVA. The correlation between SE-CFUs and SE-specific antibody titers was analyzed using the Spearman’s rank correlation test. The procedure to analyze the microbiota composition using the microarray chip has been described elsewhere [31]. Correlations between immune parameters and microbial taxa were analyzed using the Pearson product-moment correlation procedure. All statistical analyses on immunology data were performed using GraphPad Prism 9 software (GraphPad Software, CA, USA) and on microbiota data using JMP Genomics 9 software (SAS Institute 2017, NC, USA). A *p* value of  < 0.05 was considered statistically significant and a value of 0.05  <  *p*  <  0.1 is referred to as a trend, in case the *p *value did not belong to one of these categories the difference observed is referred to as “numerical”. Significant differences between groups are depicted by “*” and within a group in course of time by “#”. Microarray standardized LS means of fluorescence intensities were compared using false discovery rate (FDR) adjusted *p* values set at  < 0.05.

## Results

### GM supplementation resulted in numerically lower CFUs in some of the chickens

SE counts were not observed in the ileum, nor in the spleen of chickens fed either a standard or a GM supplemented diet before SE inoculation (Table 2). After inoculation, the number of chickens in which SE was detected in the ileum did not differ between the GM and the control group. In the GM group, SE was detected in the ileum in one out of six chickens at 14 dpi, and also in one out of six chickens in the control group at 7 dpi (Table 2). In the spleen at 7 dpi, SE was detected in three out of six chickens in the GM group compared to five out of six chickens in the control group (Table 2). Although no significant differences in SE-CFUs were observed, the average SE-CFUs was numerically lower at 7 dpi in the spleens of chickens which received a GM supplemented diet compared to the control group (Figure 1A). At 14 dpi, SE was detected in the spleen in five out of six chickens in the GM group compared to three out of six chickens in the control group (Table 2). At 21 dpi, SE was detected in the spleen in one out of six chickens in both groups (Table 2). In course of time, CFUs in the spleen decreased between 7 and 21 dpi in both groups (Figure 1A).

**Table 2:** **Number of chickens positive for SE in ileum and spleen**.

	SE presence in ileum		SE presence in spleen	
Dpi	Control	GM	Control	GM
−4	0/6	0/6	0/6	0/6
0	0/6	0/6	0/6	0/6
7	1/6	0/6	5/6	3/6
14	0/6	1/6	3/6	5/6
21	0/6	0/6	1/6	1/6

Number of chickens with positive *Salmonella* counts in the ileum and spleen, *n * = 6 per group and time point.

GM supplementation during SE infection did not affect growth performance of the chickens, as body weights were similar in chickens of control and GM groups (Figure 1B). After SE inoculation, numbers of IELs were numerically higher at 14 dpi in the GM group as compared to the control group (Figure 1C). In the spleen, numbers of leukocytes post-infection were not significantly different between the GM and the control group (Figure 1D).

### GM supplementation resulted in significantly higher numbers and an increase in activation of intraepithelial NK cells post SE infection

**Figure 2:**
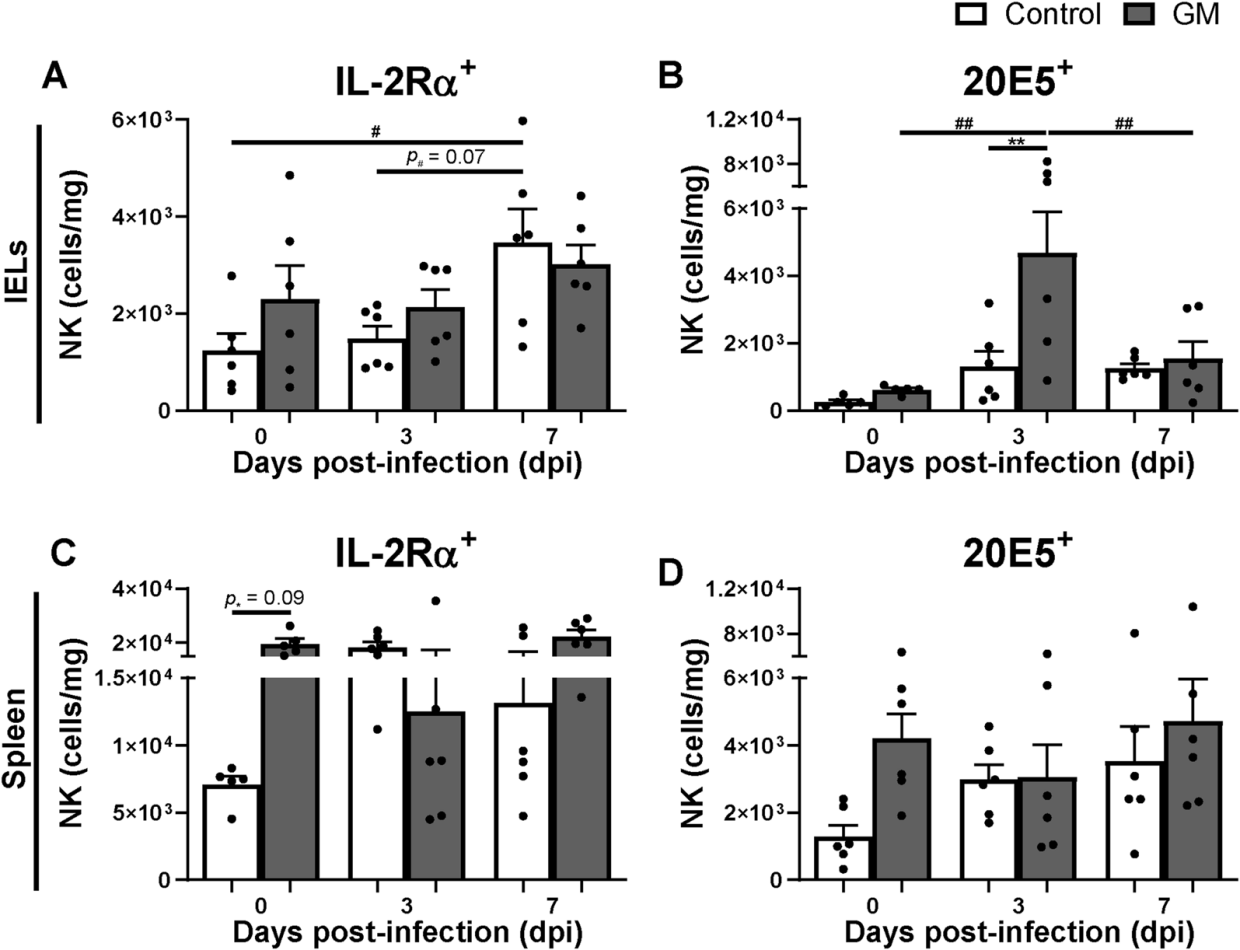
**Effect of GM on numbers of intraepithelial and splenic NK cells before and during SE infection in broiler chickens. A** Numbers (cells/mg) of intraepithelial IL-2Rα^+^ and **B** 20E5^+^ NK cells in chickens either fed standard (control) or long-chain glucomannan supplemented (GM) diet in course of time before and during SE infection. **C** Numbers (cells/mg) of splenic IL-2Rα^+^ and **D** 20E5^+^ NK cells in chickens either fed standard or GM diet before and during SE infection. Mean + SEM per diet group and time point are shown (*n*  = 6), if *n*  = 5; one chicken was excluded due to numbers of events acquired in the gate of interest were  < 100. Statistical significance between diet groups is indicated as **(*p*  < 0.01) and in course of time within a group as ^#^(*p*  < 0.05), ^##^(*p*  < 0.01).

The effect of GM supplementation on numbers of intraepithelial and splenic NK cell subsets was determined during SE infection. Post SE infection, intraepithelial IL-2Rα^+^ NK cells were numerically higher and 20E5^+^ NK cell numbers significantly higher at 3 dpi in the GM group compared to numbers in the control group (Figures 2A, B). In course of time, numbers of intraepithelial IL-2Rα^+^ NK cells in the GM group remained similar before and during infection, whereas numbers in the control group significantly increased post-infection compared to 0 dpi (Figure 2A). Numbers of intraepithelial 20E5^+^ NK cells significantly increased at 3 dpi compared to 0 dpi in the GM group only (Figure 2B). In the spleen, numbers of IL-2Rα^+^ and 20E5^+^ NK cells did not significantly differ between groups post-infection (Figures 2C, D). In course of time, splenic IL-2Rα^+^ and 20E5^+^ NK cells numerically increased post-infection compared to 0 dpi in the control group but not in the GM group (Figures 2C, D).

**Figure 3:**
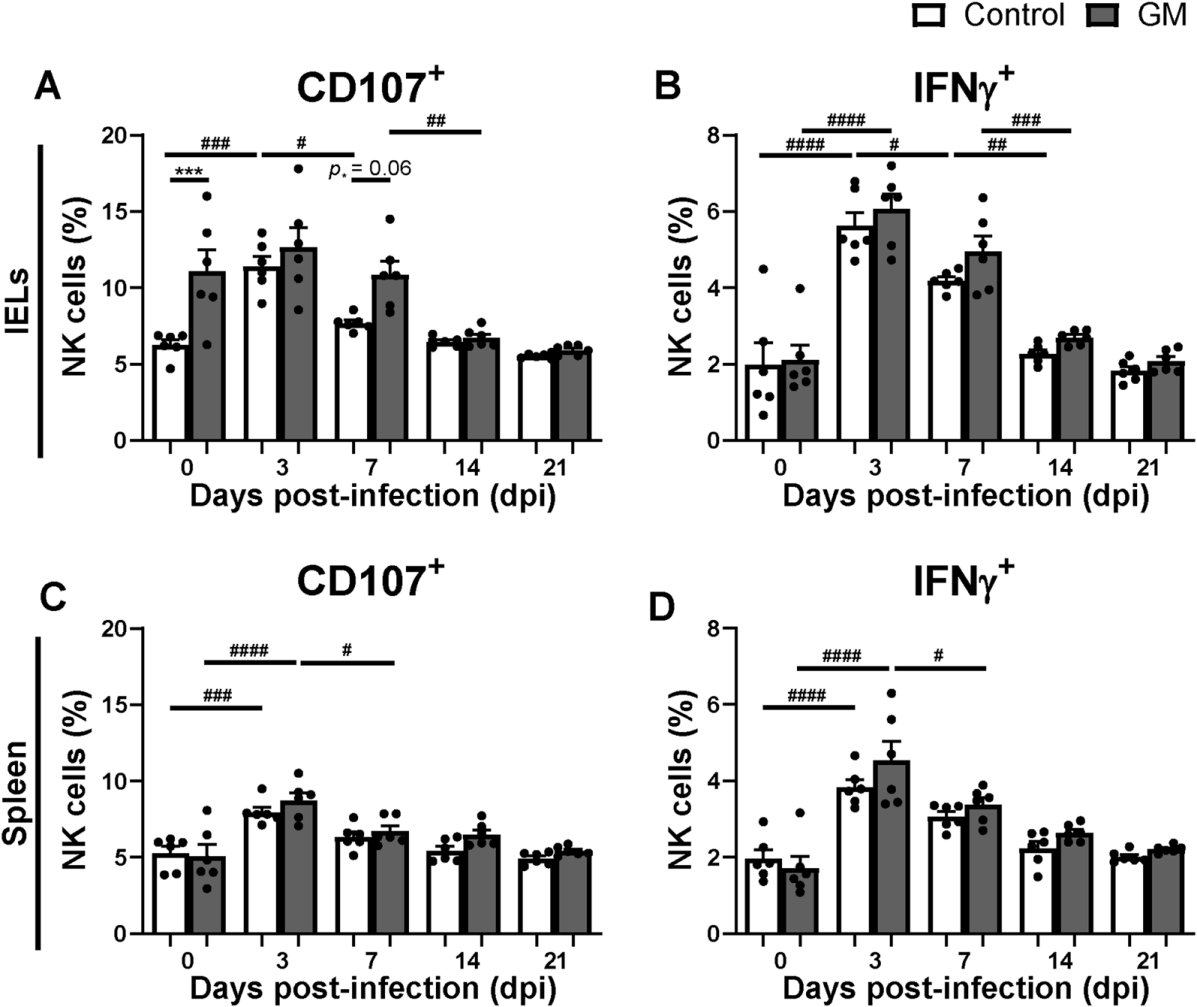
**Effect of GM on NK cell activation in IELs and spleen before and during SE infection in broiler chickens. A** Percentages of intraepithelial NK cells expressing CD107 and **B** IFNγ in chickens either fed standard (control) or long-chain glucomannan supplemented (GM) diet in course of time before and during SE infection. **C** Percentages of splenic NK cells expressing CD107 and **D** IFNγ in chickens either fed standard or GM diet before and during SE infection. Mean  +  SEM per diet group and time point are shown (*n*  = 6) and statistical significance between diet groups is indicated as ***(*p*  < 0.001) and in course of time within a group as ^#^(*p*  < 0.05), ^##^(*p*  < 0.01), ^###^(*p*  < 0.001) and ^####^(*p*  < 0.0001).

To investigate possible changes in NK cell activation due to GM supplementation during SE infection, surface expression of CD107 and intracellular expression of IFNγ were determined in NK cells isolated from the IEL population and spleen. Post SE infection, CD107 expression on intraepithelial NK cells tended to be higher at 7 dpi in the GM group compared to the control group (Figure 3A). No significant differences in IFNγ expression in intraepithelial NK cells were observed between the GM and control group during SE infection (Figure 3B). In the spleen, CD107 expression (Figure 3C) and IFNγ expression (Figure 3D) of NK cells did not differ between the groups during SE infection. In course of time, CD107 expression on intraepithelial NK cells increased at 3 dpi compared to 0 dpi in both groups, although only significantly in the control group, and then decreased until 21 dpi to the level of 0 dpi in the control group (Figure 3A). Furthermore, IFNγ expression in intraepithelial and splenic NK cells and CD107 expression of splenic NK cells significantly increased at 3 dpi compared to 0 dpi in both groups and then decreased until 21 dpi to levels before infection (Figures 3B–D).

### GM supplementation resulted in an increase of intraepithelial cytotoxic CD8^+^ T cell numbers post SE infection

**Figure 4:**
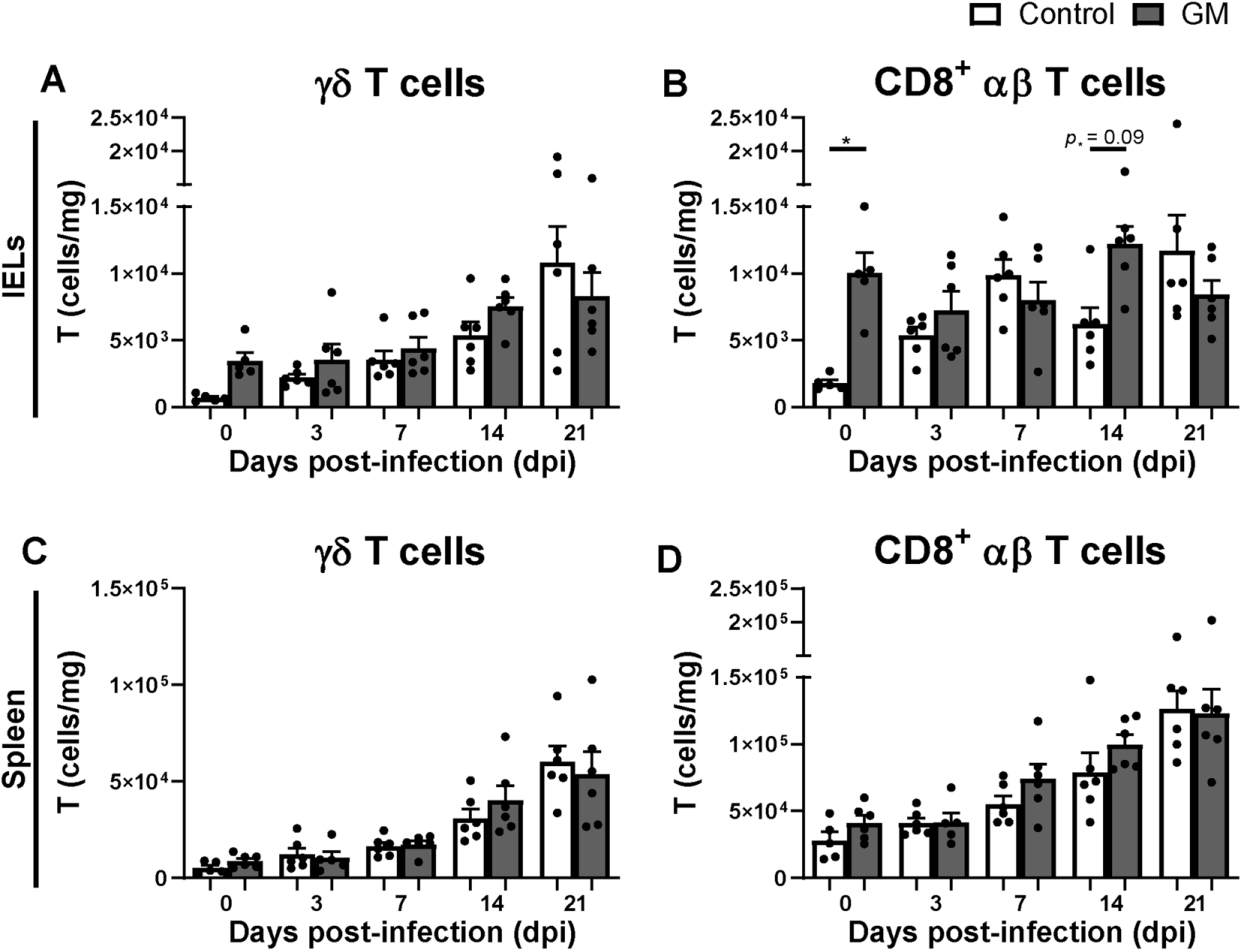
**Effect of GM on numbers of intraepithelial and splenic T cells before and during SE infection in broiler chickens. A** Numbers (cells/mg) of intraepithelial γδ T cells and **B** CD8^+^ αβ T cells in chickens either fed standard (control) or long-chain glucomannan supplemented (GM) diet in course of time before and during SE infection. **C** Numbers (cells/mg) of splenic γδ T cells and **D** CD8^+^ αβ T cells in chickens either fed standard or GM diet before and during SE infection. Mean  +  SEM per diet group and time point are shown (*n*  = 6), if *n*  = 5; one chicken was excluded due to numbers of events acquired in the gate of interest were  < 100. Statistical significance between diet groups is indicated as *(*p*  < 0.05).

The effect of GM supplementation on numbers of intraepithelial and splenic γδ T cells and cytotoxic (CD8^+^) αβ T cells was investigated during SE infection. Post SE infection, intraepithelial γδ T cells were numerically higher and numbers of cytotoxic CD8^+^ T cells tended to be higher at 14 dpi in the GM group compared to the control group (Figures 4A, B). In course of time, intraepithelial γδ T cells numerically increased post-infection compared to 0 dpi in both groups. Numbers of intraepithelial cytotoxic CD8^+^ T cells remained similar post-infection compared to 0 dpi in the GM group, whereas these cells numerically increased during this period in the control group (Figures 4A, B). In the spleen, no differences in numbers of γδ T cells (Figure 4C) and cytotoxic CD8^+^ T cells (Figure 4D) were observed between the GM and control group during SE infection. In course of time, γδ T cells and cytotoxic CD8^+^ T cells numerically increased post-infection compared to 0 dpi in both groups (Figures 4C, D).

Next, γδ T cells and cytotoxic αβ T cells were analyzed for their CD8αα and CD8αβ expression. Both CD8αα^+^ and CD8αβ^+^ intraepithelial γδ T cells were numerically higher at 14 dpi in the GM group compared to the control group (Additional files 2A and B). Intraepithelial cytotoxic CD8αα^+^ T cells were numerically higher and CD8αβ^+^ T cell numbers were significantly higher at 14 dpi in the GM group compared to the control group (Additional files 2C and D). In course of time, CD8αα^+^ and CD8αβ^+^ intraepithelial γδ T cells numerically increased post-infection compared to 0 dpi in both groups. Numbers of CD8αα^+^ and CD8αβ^+^ intraepithelial cytotoxic T cells remained similar post-infection compared to 0 dpi in the GM group, whereas these cells numerically increased during this period in the control group (Additional files 2A–D). In the spleen, no differences in numbers of CD8αα^+^ and CD8αβ^+^ γδ T cells nor cytotoxic CD8αα^+^ and CD8αβ^+^ T cells were observed between the GM and control group during SE infection. In course of time, these splenic T cells numerically increased post-infection compared to 0 dpi in both groups (Additional files 2E–H).

Finally, possible changes in T cell activation upon GM supplementation during SE infection were determined. Post SE infection in the IELs and spleen, no differences in CD107 expression on CD8^+^ T cells (comprising both γδ and αβ TCRs) nor IFNγ expression of CD8^+^ γδ T cells, cytotoxic CD8^+^ T cells and CD4^+^ T cells (spleen only) between the GM and control group were observed (Additional file 3).

### GM supplementation resulted in a higher SE-specific antibody response

**Figure 5:**
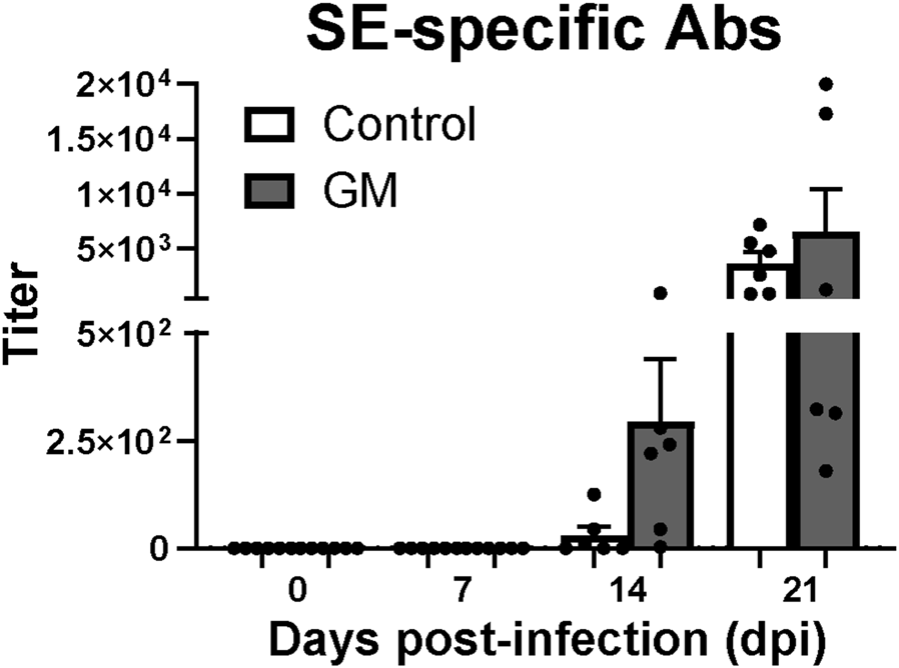
**Effect of GM on serum antibody titers as a response to SE infection in broiler chickens.** Titers of SE-specific antibodies in sera of chickens either fed standard (control) or long-chain glucomannan supplemented (GM) diet in course of time before and during SE infection. Mean  +  SEM per diet group and time point are shown (*n*  = 6).

The effect of GM supplementation on titers of SE-specific antibodies in the serum was determined in course of time post SE infection. Although no significant differences were observed between the groups, all six chickens in the GM group showed SE-specific antibody titers at 14 dpi, which were numerically higher compared to antibody titers of the three out of six chickens in the control group (Figure 5). At 21 dpi, all chickens showed SE-specific antibody responses and titers were numerically higher in the GM group compared to the control group (Figure 5). In addition, a significant negative correlation between the antibody titers and SE-CFUs in the spleen was observed although it was weak (r  = −0.41, Additional file 4).

### GM supplementation resulted in significantly higher relative abundance of lactic acid bacteria in microbiota of the ileum and caeca post SE infection

**Figure 6:**
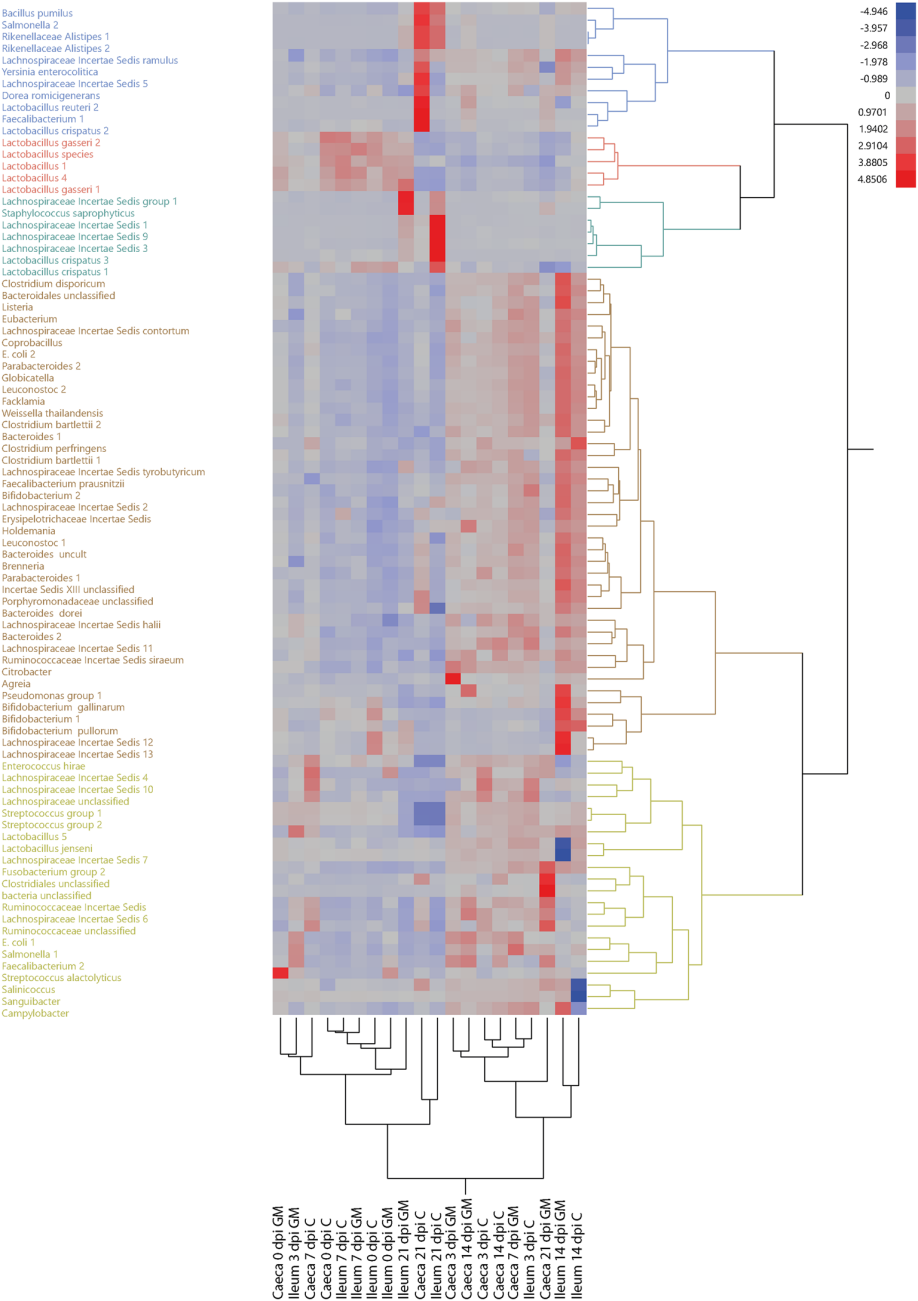
**Effect of GM on microbiota composition in ileum and caeca before and during SE infection in broiler chickens.** Hierarchical cluster analysis of relative abundance of microbial taxa targeted by the microarray in the ileum and caeca of broiler chickens either fed standard (C) or long-chain glucomannan supplemented (GM) diet at 0, 3, 7, 14 and 21 days post SE infection. The standardized relative fluorescence intensities of the microarray are depicted in a heatmap as low (blue) or high (red) relative abundance of microbial taxa. Microbiota clustered first by intestinal segment in ileum (left) or caeca (right) and subsequently by age in five clusters, but this clustering was affected by diet. Microbial taxa are colored by cluster (blue, red, green, brown, yellow). Per intestinal segment, age and diet *n*  = 6 and with statistical significance of FDR adjusted *p *values set at  < 0.05.

Changes in the microbiota composition of the ileum and caeca due to GM supplementation during SE infection were determined using a microarray. Microbial analysis of standardized LS means as a parameter for relative abundance among the variables intestinal segment (caeca/ileum), age (dpi), diet (Control/GM) and their three-way interaction, revealed a total of 86 bacterial taxa identified by the probes that were significantly different. By two-way hierarchical clustering between variables (clustered vertically) and significantly different bacterial taxa (clustered horizontally), microbiota profiles were divided first in two clusters (Figure 6). In the left cluster, 7 out of the 11 profiles were samples from the ileum while in the right cluster 6 out of the 9 profiles were samples from the caeca. This indicates that with few exceptions, the variable intestinal segment was important to define clustering of microbiota profiles by similarity. Within these two clusters, further clustering seems to group microbiota profiles based on age and then diet.

Factorial interaction analysis of the relative abundance of microbial taxa showed differences in microbiota compositions post SE infection of which more *Faecalibacterium* 2 was detected in both ileum and caeca in the GM group, whereas more *Lachnospiraceae* 10 was observed in the caeca of chickens in the control group at 3 dpi (Table 3; Additional file 5). At 7 dpi, no differences were found in microbiota composition of the ileum between the two diet groups, however, in the caeca, higher levels of *Salmonella* probe 1 were detected in the GM group (Table 3; Additional file 5).

**Table 3:** **Microbial taxa that showed significant higher relative abundance with either the control or GM feed before and during SE infection**.

Dpi/intestinal segment	Control	GM
0 dpi/ileum	–	–
0 dpi/caeca	–	*Streptococcus alactolyticus*
3 dpi/ileum	–	*Faecalibacterium* 2
3 dpi/caeca	*Lachnospiraceae* Incertae Sedis 10	*Agreia*, *Faecalibacterium* 2
7 dpi/ileum	–	–
7 dpi/caeca	–	*Salmonella* 1
14 dpi/ileum	*Lactobacillus jenseni*, *Lachnospiraceae* Incertae Sedis 7	*Bifidobacterium gallinarium*, *Sanguibacter*, *Lachnospiraceae* Incertae Sedis 12 and 13, *Salinococcus*, *Campylobacter*
14 dpi/caeca	–	–
21 dpi/ileum	*Lactobacillus crispatus* 3, *Lachnospiraceae* Incertae Sedis 1, 3 and 9, *Rikenellaceae Alistipes* 1 and 2, *Salmonella* 2	*Streptococcus* group 1 and 2, *Lactobacillus gasseri* 1, *Lactobacillus* 4, *Lactobacillus* sp.
21 dpi/caeca	*Rikenellaceae Alistipes* 1 and 2, *Faecalibacterium* 1, *Lactobacillus crispatus* 2, *Lactobacillus reuteri* 2, *Lachnospiraceae* Incertae Sedis 5, *Yersina enterocolitica*, *Bacillus pumilus*, *Dorea formicigenerans*, *Salmonella* 2	*Streptococcus* group 1 and 2, *Enterococcus hirae*, *Faecalibacterium* 2, *Fusobacterium* group 2, *Ruminococcaceae* unclassified, *Clostridiales* unclassified, Bacteria unclassified

The intestinal bacterial taxa identified by the probes of which the standardized LS means of fluorescence intensity was significantly higher in chickens given the respective feed, as determined by factorial analysis of pairwise comparisons between feed groups before and during SE infection in the ileum or the caeca of broiler chickens. Feed groups included standard diet (control) and long-chain glucomannan supplemented diet (GM), with statistical significance of FDR adjusted *p *values set at  < 0.05.

**Figure 7:**
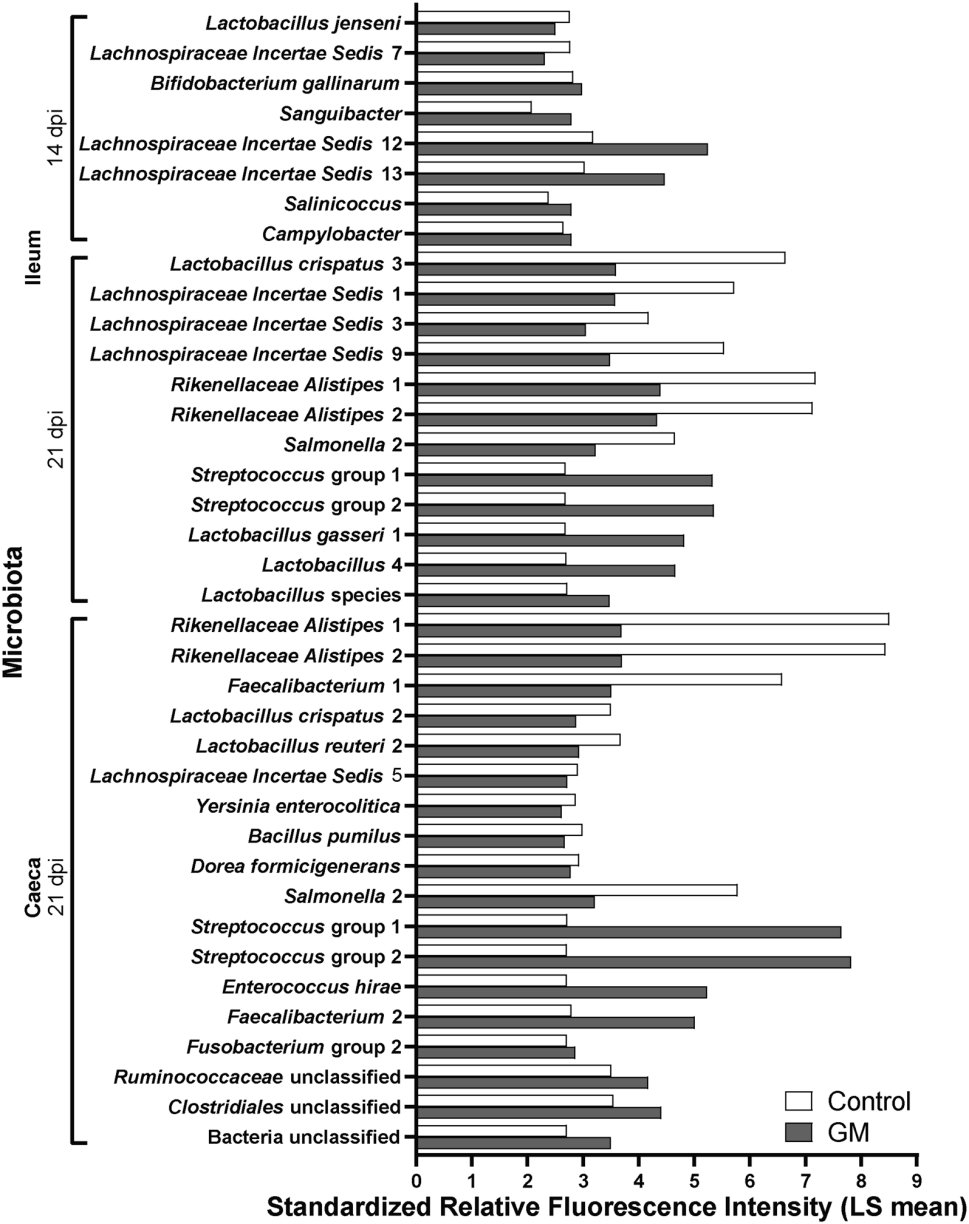
**Intestinal microbial taxa significantly increased with diet at 14 and 21** **dpi of SE in broiler chickens.** Standardized relative fluorescence intensities of the microbial taxa as measured by the microarray in the ileum and caeca (Table 3) that were significantly higher either with standard (control) or long-chain glucomannan supplemented (GM) diet at 14 and 21 dpi of SE in broiler chickens. LS mean per microbial taxa and diet group are shown (*n*  = 6) with statistical significance of FDR adjusted *p *values set at  < 0.05.

**Figure 8:**
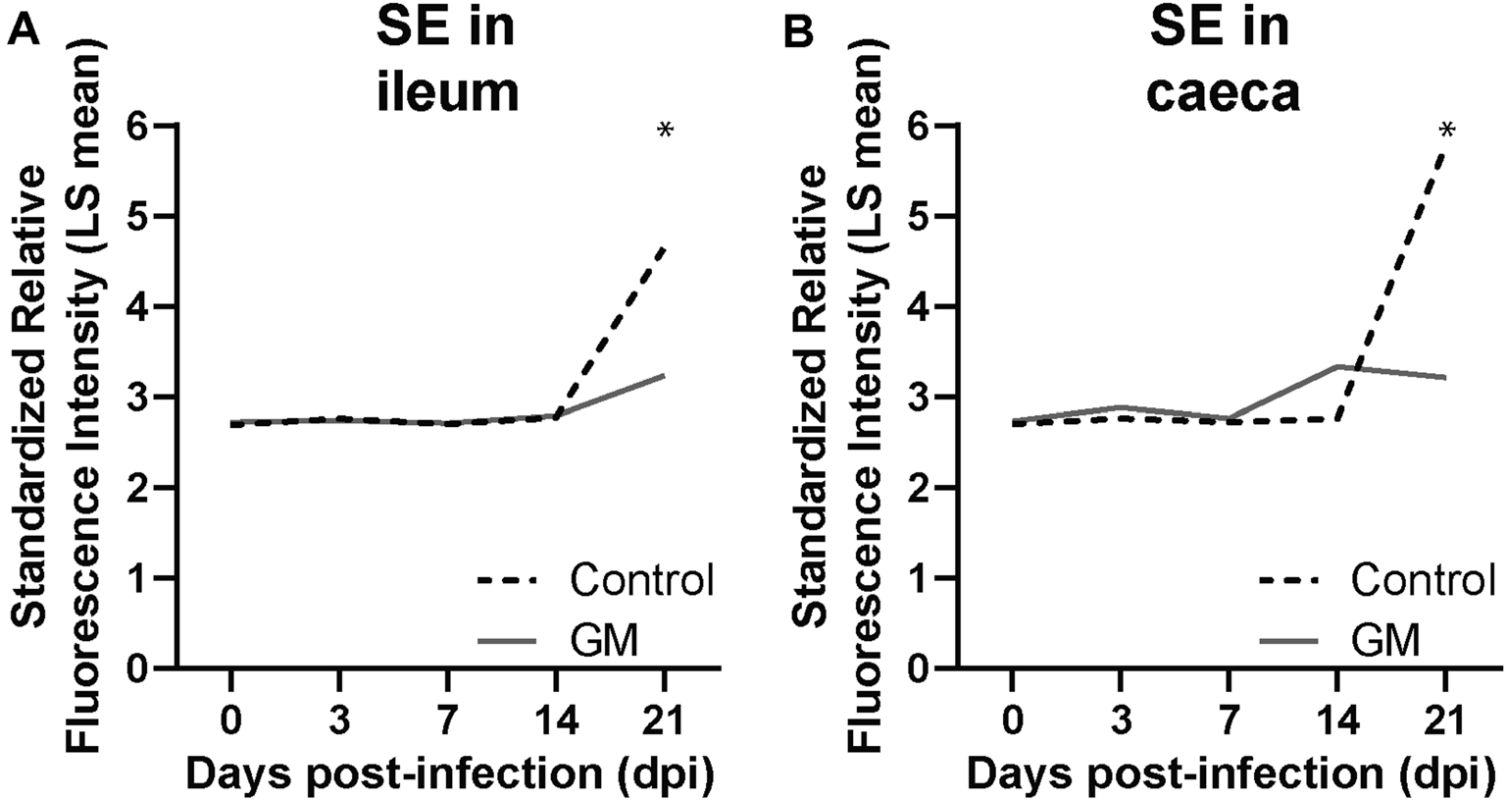
**Effect of GM on relative abundance of SE in the microbiota of ileum and caeca before and during SE infection in broiler chickens. A** Standardized relative fluorescence intensities of SE (*Salmonella* probe 2) as measured by the microarray in microbiota of the ileum and **B** caeca of chickens either fed standard (control) or long-chain glucomannan supplemented (GM) diet in course of time before and during SE infection. LS mean per diet group and time point are shown (*n*  = 6) and statistical significance between diet groups is indicated as *(FDR adjusted *p *values set at  < 0.05).

The significant differences in microbial taxa between the control and GM group were more evident in chickens at 14 and 21 dpi, where GM supplementation induced higher relative abundance of LAB including *Lactobacillus*, *Bifidobacterium*, *Streptococcus* and *Enterococcus* species. At 14 dpi, more *Bifidobacterium gallinarium* and *Lachnospiraceae* 12 and 13 were observed in the GM group compared to more *Lactobacillus jenseni* and *Lachnospiraceae* 7 in the ileum of chickens in the control group (Table 3; Figure 7). No differences in microbiota composition between groups were found in the caeca at 14 dpi (Table 3). At 21 dpi, several streptococci*, Lactobacillus gasseri* 1 and *Lactobacillus* 4 showed a higher relative abundance in the GM group, whereas more *Lactobacillus crispatus* 3, *Lachnospiraceae* 1, 3 and 9, and *Salmonella* probe 2 were detected in the ileal microbiota in the control group (Table 3; Figure 7). In the caecal microbiota of the GM group, more streptococci, *Enterococcus hirae*, *Faecalibacterium* 2 and *Fusobacterium* group 2 were found, compared to more *Faecalibacterium* 1, *Lactobacillus crispatus* 2, *Lactobacillus reuteri* 2 and *Lachnospiraceae* 5 in the control group (Table 3; Figure 7). In course of time towards 21 dpi, the relative abundance of SE (*Salmonella* probe 2) significantly increased in the ileum (Figure 8A) and caeca (Figure 8B) of chickens fed the standard diet compared to GM supplemented diet.

### Relative abundance of lactic acid bacteria correlate positively with NK cell activation and SE-specific antibodies, and negatively with SE-CFUs

**Figure 9:**
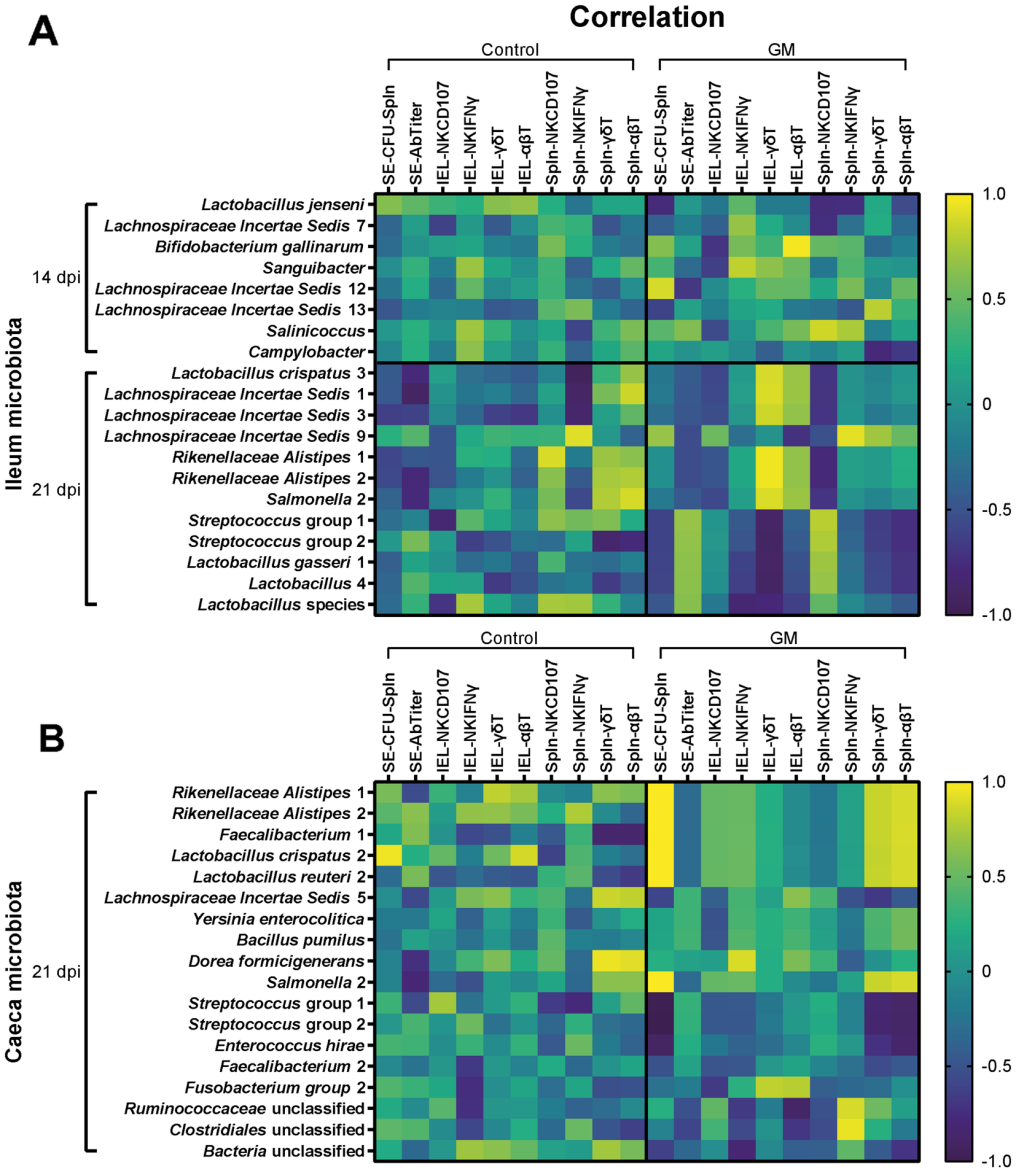
**Correlation between microbial taxa and intraepithelial and splenic immune parameters at 14 and 21** **dpi of SE in broiler chickens. A** Correlation values between intestinal microbial taxa in the ileum or **B** caeca significantly increased with diet and splenic SE-CFUs, serum antibody titers, percentages of NK cell activation (CD107 or IFNγ expression) or numbers of NK and T cell subsets of the ileum (IEL) and spleen (Spln) per diet (control, GM) at 14 and 21 dpi of SE in broiler chickens. Pearson’s correlation ® values are depicted in a heatmap as positive (yellow) or negative (dark blue) correlations.

Since GM supplementation affected immune parameters and microbiota composition during SE infection, we analyzed whether these effects were related by performing a Pearson’s correlation analysis. Positive and negative correlations were reported between significantly higher microbial taxa mentioned in Table 3, and SE-CFUs, SE-specific antibody titers, NK cell CD107 expression, NK cell IFNγ expression, NK and T cell subsets in the IEL population and spleen for the respective intestinal segment, diet and age (Figure 9 and Additional file 6). Based on our observations that GM supplementation significantly increased relative abundance of LAB, the correlations between LAB and immune parameters are highlighted. At 3 dpi, no strong correlations were observed for ileal microbiota in both groups (Additional file 6A). For the caecal microbiota early post SE infection (0–7 dpi), strong positive and negative correlations were observed of bacteria other than LAB (other bacteria) with intraepithelial NK cell subsets and splenic γδ T cells in both groups (Additional file 6B). Most differences in the microbiota of ileum and caeca and strongest correlations were observed at 14 and 21 dpi (Figure 9). At 14 dpi in the ileal microbiota, strong correlations between immune parameters and microbial taxa in the GM group were found in contrast to no strong correlations in the control group (Figure 9A). In the GM group, strong positive correlations were observed between intraepithelial cytotoxic αβ T cells and *Bifidobacterium gallinarum*, and of several other bacteria with SE-CFUs, intraepithelial IFNγ^+^ NK cells and splenic CD107^+^ NK cells and γδ T cells (Figure 9A).

At 21 dpi in the ileal microbiota in the GM group, a pattern in the correlations was observed with bacteria that had a lower (*Lactobacillus crispatus* 3 and other bacteria) or higher (bottom five LAB taxa) relative abundance compared to the control group (Figure 9A). *Lactobacillus crispatus* 3 and other bacteria showed strong positive correlations with intraepithelial γδ T cells and moderate negative correlations with splenic CD107^+^ NK cells. In contrast, LAB including streptococci in the ileal microbiota in the GM group at 21 dpi showed strong negative correlations with intraepithelial γδ T cells and moderate positive correlations with SE-specific antibody titers and splenic CD107^+^ NK cells (Figure 9A). In ileal microbiota in the control group at 21 dpi, *Lactobacillus crispatus* 3 and other bacteria showed strong negative and positive correlations with SE-specific antibody titers, intraepithelial and splenic CD107^+^ and IFNγ^+^ NK cells and splenic T cell subsets (Figure 9A). In the caecal microbiota in the GM group at 21 dpi, a pattern in the correlations was observed again with bacteria that had a lower (two *Lactobacillus* species and other bacteria) or higher (bottom eight taxa) relative abundance compared to the control group (Figure 9B). The two *Lactobacillus* species and several other bacteria showed strong positive correlations with SE-CFUs and splenic γδ T cells and cytotoxic αβ T cells. In contrast, LAB including streptococci in the caecal microbiota at 21 dpi showed strong negative correlations with SE-CFUs and splenic γδ T cells and cytotoxic αβ T cells (Figure 9B). In the caecal microbiota in the control group at 21 dpi, strong positive and negative correlations were observed of *Lactobacillus crispatus* 2 and other bacteria with SE-CFUs and intraepithelial and splenic T cell subsets (Figure 9B).

## Discussion

In the current study, we investigated the protective potential of long-chain glucomannan supplementation against experimental SE exposure in seven day old broiler chickens. The chickens were successfully infected with SE as was demonstrated by the presence of bacteria in ileum, caeca and spleen and the detection of SE-specific antibodies. At 7 dpi, SE was detected in a lower number of chickens in the GM supplemented group, moreover the average CFU was numerically lower in the spleen of these chickens as compared to the animals fed the standard diet. In addition, SE counts in the spleen decreased between 7 and 21 dpi in both groups. Although numbers of SE in the spleen had increased at 14 and 21 dpi in the GM group, highest SE counts in course of infection were detected in the control group. These observations led to the hypothesis that the kinetics of SE during infection may be associated with GM supplementation but further investigation of the causal mechanisms is needed. No differences in SE counts were found between groups upon plating of total cell suspensions from ileum. A significant difference in the relative abundance of SE was observed at 21 dpi resulting from the microarray analysis of ileal and caecal microbiota of chickens that received the GM supplemented diet compared to the standard diet. These data show a different dynamic in SE levels compared to the expectation of a gradual increase of SE upon inoculation. These observations also imply the importance of both sample type and method for the detection of SE. The observed immune responses to SE in the standard diet group are similar to those of a previously conducted study that investigated innate and adaptive immune responses in the course of SE infection in comparison to uninfected chickens [17]. Furthermore, the results are in agreement with a study showing lower presence of *Salmonella enterica* serotype Typhimurium (ST) in the intestine and liver of chickens due to galactoglucomannan supplementation [40]. The lower numbers of SE in the spleen and reduced SE colonization of the intestine indicates that GM supplementation may impact SE infection of young broiler chickens. Several mechanisms could explain the effect of GM supplementation on SE infection.

First, GM supplementation may affect SE due to stimulation of the immune system. GM supplemented chickens indeed showed increased numbers and activation of intraepithelial NK cells at respectively 3 and 7 dpi, as compared to chickens receiving the standard diet. A distinct population of IL-2Rα^+^ 20E5^+^ cells was not observed, although the possibility that these cells exist at a very low frequency cannot be excluded. The stimulatory impact of GM affecting numbers and function of intraepithelial NK and T cells before infection is similar to observations in uninfected chickens of the same age supplemented with GM [31]. Whether the distinct increase in 20E5^+^ intraepithelial NK cells is a result of recruitment of 20E5^+^ NK cells or development of IL-2Rα^+^ into 20E5^+^ NK cells needs to be investigated in future experiments. GM supplementation enhanced expression of CD107 on intraepithelial NK cells but not IFNγ expression before infection, and a similar trend was observed for intraepithelial CD8^+^ T cells. Early post-infection IFNγ expression in NK cells was enhanced equally in both diet groups, which was similar to our previous study with SE-infected chickens fed a standard diet [17]. This suggests that GM results in increased responsiveness before and during the start of SE infection by stimulation of degranulation rather than IFNγ production as effector pathways of these cells. Furthermore, GM supplementation was associated with stronger subsequent adaptive immune responsiveness, since numbers of intraepithelial cytotoxic CD8^+^ T cells and SE-specific serum antibody responses were higher at 14 dpi compared to the standard diet. The higher number of activated NK cells in chickens upon GM supplementation are likely to have contributed to these subsequent responses by secretion of cytokines and chemokines that promote interaction between antigen-presenting cells and cells of the adaptive immune system, resulting in higher antibody levels [52]. Higher numbers of intraepithelial CD8αα^+^ and CD8αβ^+^ cytotoxic T cells were observed in GM supplemented chickens compared to the standard diet post-infection, although only significant in the CD8αβ^+^ subset, suggesting a role in the defense against SE. Interestingly, the stimulatory effects of GM on the numbers of intraepithelial T cells is stronger under SE challenge as compared to non-challenged GM-supplemented chickens [31]. The stimulatory effects of GM on NK and T cells may be facilitated by the cytosolic aryl hydrocarbon receptor (AHR) or G-protein coupled receptors (GPCRs) that recognize diet-derived ligands as was shown in humans and mice [53–55]. Our data are in line with studies that describe the effect of other polysaccharides such as yeast glucans and prebiotics, which were shown to reduce SE colonization and invasion by upregulated expression of intestinal innate-immunity-related genes [41, 43, 56], enhanced killing of SE by macrophages [57], and increased SE-specific intestinal IgA and serum IgG antibody responses [43]. Future research should address which specific characteristics of GM lead to the observed increased immune responsiveness.

A second mechanism of GM supplementation that may have an effect on SE is modulation of the intestinal microbiota composition, resulting in microbiota profiles different from the ones in chickens fed the standard diet during a SE infection. Differences in relative abundance of microbial taxa between the chickens in the two diet groups increased with time post-infection. GM supplemented chickens showed increased relative abundance of lactic acid bacteria (LAB) including *Streptococcus* species, *Lactobacillus* species and *Bifidobacterium gallinarum* in the ileum and caeca compared to the ones fed the standard diet. This observation was in agreement with a previous study using GM supplementation [31] and may indicate that these LAB preferentially benefit from GM. Before SE infection, the relative abundance of only Streptococci was increased in the caeca of chickens receiving GM supplementation. This finding was different from a previous observation where GM increased relative abundance of multiple LAB in the ileum and caeca of uninfected chickens at 7 days of age [31] and may result from variations in microbial composition among chickens [58]. The increased relative abundance of LAB during SE infection with GM supplementation is in agreement with other studies providing prebiotics during SE or ST infection [41, 42, 56]. The high relative abundance of streptococci was associated with the lower relative abundance of SE in both ileal and caecal microbiota at 21 dpi in chickens receiving GM supplementation compared to the standard diet, suggesting competitive exclusion. This observation is in line with another study showing that commensal LAB strains including *Enterococcus* and *Streptococcus* contribute to a low-shedder phenotype of SE-infected chickens [59]. Furthermore, these LAB strains proved to have immunomodulatory properties on innate cells in humans [60]. This is mediated by production of metabolites like short-chain fatty acids (SCFAs), which can function as energy substrate for the host and microbes [61, 62], but are also known to affect intestinal NK and T cell function in support of maintenance of intestinal homeostasis [61, 63–65]. Production of SCFAs by LAB and other microorganisms able to use lactic acid as a substrate for SCFA production was shown previously to be increased during ST infection in chickens [61]. In addition, the production of organic acids such as lactic acid by *Lactobacillus* species may contribute to the reduction of SE, since these were shown to inhibit growth of SE and ST in vitro through acidification of the environment [66]. These findings indicate that GM supplementation results in a lower presence of SE most likely by competitive exclusion, microbial metabolites or indirect stimulation of innate immune cells via the microbiota.

Finally, we addressed the potential interference of the effects of GM supplemented diet between immune parameters and microbiota during SE infection. A negative correlation between relative abundance of commensal streptococci and numbers of SE in the spleen at 21 dpi was revealed. Furthermore, positive correlations were found between relative abundance of *Bifidobacterium gallinarum* and numbers of intraepithelial cytotoxic T cells at 14 dpi, and between relative abundance of streptococci and splenic NK cell activation as well as SE-specific antibody responses at 21 dpi. In addition, GM-related positive correlations were observed between relative abundance of Streptococci and intraepithelial NK cell activation as well as numbers of splenic IL-2Rα^+^ NK cells before SE infection, in agreement with previous findings of correlations between LAB and immune cells [31]. These correlates indicate involvement of LAB in the recruitment and functioning of immune cells that may contribute to the outcome of SE in GM supplemented chickens. GM is suggested to exert its effects indirectly on NK cells, mainly before, and on T cells, mainly post-infection, by local interactions with LAB or their metabolites in the intestine. Moreover, GM may have systemic effects on those immune cells and antibody responses, due to translocation of microbial products into the circulation or a yet hypothetical interaction with antigen presenting cells that have interacted with intestinal LAB [67, 68].

Another mechanism that can explain the observed reduction in relative abundance of SE in the intestine upon GM supplementation involves the direct binding to SE of the mannose within the long-chain glucomannan, which thereby reduces attachment, hence, colonization of SE in the intestine of broiler chickens [28].

In conclusion, supplementation of long-chain glucomannan stimulated recruitment and function of NK and T cells, the relative abundance of LAB in the intestinal microbiota as well as the interaction between these, coinciding with a reduction of SE counts in the first week post-infection in broiler chickens. Although chickens fed the GM supplemented diet still became infected with SE, less chickens were SE positive, the SE counts were lower at 7 dpi and the relative abundance of SE was lower in the intestine at 21 dpi compared to chickens fed a standard diet. As a consequence, the lower SE colonization may also lead to reduced spread of SE within a flock and hence reduced food safety risks for humans. Future studies should investigate effects of providing the GM diet on spreading of SE from infected to uninfected chickens and flock prevalence of SE at time of slaughter to test this hypothesis. Furthermore, for practical application of GM future studies should include larger scale trials and detailed monitoring of performance. The present study provides evidence for the potential to use long-chain glucomannan in practice to stimulate both the immune system and intestinal LAB and affect SE infections in young chickens.

## Supplementary Information

**Additional file 1. The gating strategies used to analyze numbers and function of NK cells, γδ T cells and cytotoxic CD8**^**+**^** T cells in the ileum.** Gating included consecutive selection for lymphocytes (FSC-A vs SSC-A), viable cells (Live/Dead marker-negative) followed by selection of the specific cellular subsets and the expression of activation markers by NK and T cells according to the staining panels (Table 1). NK cell subsets were gated on CD3^−^ cells expressing either IL-2Rα or 20E5 and NK cell activation was gated on CD3^−^CD41/61^−^ cells expressing CD107 or on CD3^−^ cells expressing IFNγ. T cell subsets were gated on CD3^+^CD4^−^ cells positive for TCRγδ (γδ) or negative (CD8^+^ αβ) with both γδ and cytotoxic αβ T cells expressing either CD8αα or CD8αβ. T cell activation was gated on CD3^+^CD41/61^−^CD8α^+^ cells expressing CD107 or on CD3^+^TCRγδ^+^CD8α^+^ and CD3^+^TCRγδ^−^CD8α^+^ cells expressing IFNγ, and only in spleen also CD3^+^TCRγδ^−^CD8α^−^ (CD4^+^) cells expressing IFNγ. The marker CD41/61 is included in the CD107 assay to exclude thrombocytes from analysis, since activated thrombocytes have been reported to express CD107 [69]. In the CD107 assay NK cells are gated by excluding T cells and thrombocytes since a pan NK marker is missing while for phenotyping the NK cells are gated based on expression of the NK markers IL-2Rα and 20E5, which are known to be expressed on cells with NK function [48].

**Additional file 2. Effect of GM on numbers of intraepithelial and splenic γδ T cells and cytotoxic T cells expressing either CD8αα**^**+**^** and CD8αβ**^**+**^** before and during SE infection in broiler chickens. A** Numbers (cells/mg) of intraepithelial CD8αα^+^ γδ T cells, **B** CD8αβ^+^ γδ T cells, **C** cytotoxic CD8αα^+^ T cells and **D** CD8αβ^+^ T cells in chickens either fed standard (control) or long-chain glucomannan supplemented (GM) diet in course of time before and during SE infection. **E** Numbers (cells/mg) of splenic CD8αα^+^ γδ T cells, **F** CD8αβ^+^ γδ T cells, **G** cytotoxic CD8αα^+^ T cells and **H** CD8αβ^+^ T cells in chickens either fed standard or GM diet before and during SE infection. Mean  +  SEM per diet group and time point are shown (*n*  = 6), if *n*  = 5; one chicken was excluded due to numbers of events acquired in the gate of interest were  < 100. Statistical significance between diet groups is indicated as ***(*p*  < 0.001).

**Additional file 3. Effect of GM on T cell activation in IELs and spleen before and during SE infection in broiler chickens. A** Percentages of intraepithelial CD8^+^ T cells expressing CD107 (including both γδ and αβ T cells), **B** CD8^+^ γδ T cells expressing IFNγ and **C** CD8^+^ αβ T cells expressing IFNγ in chickens either fed standard (control) or long-chain glucomannan supplemented (GM) diet in course of time before and during SE infection. **D** Percentages of splenic CD8^+^ T cells expressing CD107 (including both γδ and αβ T cells), **E** CD8^+^ γδ T cells expressing IFNγ, **F** CD4^+^ αβ T cells expressing IFNγ and **G** CD8^+^ αβ T cells expressing IFNγ in chickens either fed standard or GM diet before and during SE infection. Mean  +  SEM per diet group and time point are shown (*n * = 6), for IFNγ expression of CD8^+^ γδ T cells in the IEL population at 0 dpi percentages were not determined (nd) due to numbers of events acquired in the gate of interest were  <  100.

**Additional file 4. Correlation between serum antibody titers and SE-CFUs in broiler chickens.** Correlation between SE-specific antibody titers and splenic SE-CFUs of chickens either fed standard (control) or long-chain glucomannan (GM) diet using the Spearman rank correlation test. Statistical significance is indicated as *p * = 0.01.

**Additional file 5. Intestinal microbial taxa significantly increased with diet at 0, 3 and 7** **dpi of SE in broiler chickens.** Standardized relative fluorescence intensities of the microbial taxa as measured by the microarray in the ileum and caeca (Table 3) that were significantly increased either with standard (control) or long-chain glucomannan supplemented (GM) diet at 0, 3 and 7 dpi of SE in broiler chickens. LS mean per microbial taxa and diet group are shown (*n*  = 6) with statistical significance of FDR adjusted *p *values set at  < 0.05.

**Additional file 6. Correlation between microbial taxa and intraepithelial and splenic immune parameters at 0, 3 and 7** **dpi of SE in broiler chickens. A** Correlation values between intestinal microbial taxa in the ileum or **B** caeca significantly increased with diet and percentages of NK cell activation (CD107 or IFNγ expression) or numbers of NK and T cell subsets of the ileum (IEL) and spleen (Spln) per diet (control, GM) at 0, 3 and 7 dpi of SE in broiler chickens. Pearson’s correlation (r) values are depicted in a heatmap as positive (yellow) or negative (dark blue) correlations.

## Data Availability

All data generated or analyzed during this study are included in this published article (and its Additional files).

## Abbreviations

SE: *Salmonella enterica* serotype Enteritidis
GM: long-chain glucomannan
dpi: days post-infection
NK cell: natural killer cell
LAB: lactic acid bacteria
IELs: intraepithelial lymphocytes
RT: room temperature
CFUs: colony-forming units
LOD: limit of detection
FDR: false discovery rate
ST: *Salmonella enterica* serotype Typhimurium
