# Supplementary figures and images for: Long-chain glucomannan supplementation modulates immune responsiveness, as well as intestinal microbiota, and impacts infection of broiler chickens with Salmonella enterica serotype Enteritidis
Source: Vet Res. 2022 Feb 4;53:9. doi: 10.1186/s13567-022-01026-z (PMC8817541; doi:10.1186/s13567-022-01026-z)

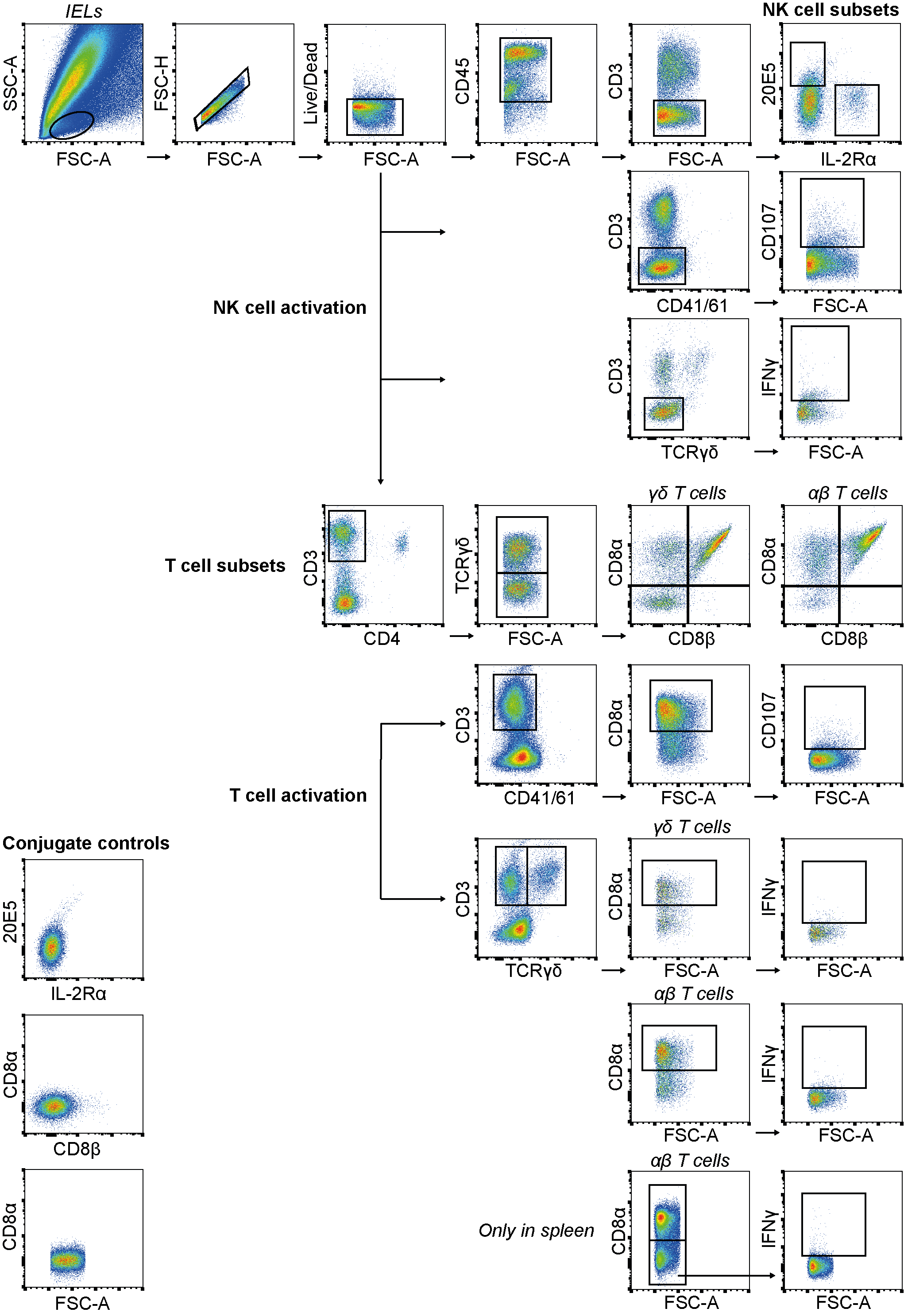

Supplement: Supplementary file 1 — Additional file 1. The gating strategies used to analyze numbers and function of NK cells, γδ T cells and cytotoxic CD8+ T cells in the ileum. Gating included consecutive selection for lymphocytes (FSC-A vs SSC-A), viable cells (Live/Dead marker-negative) followed by selection of the specific cellular subsets and the expression of activation markers by NK and T cells according to the staining panels (Table 1). NK cell subsets were gated on CD3− cells expressing either IL-2Rα or 20E5 and NK cell activation was gated on CD3−CD41/61− cells expressing CD107 or on CD3− cells expressing IFNγ. T cell subsets were gated on CD3+CD4− cells positive for TCRγδ (γδ) or negative (CD8+ αβ) with both γδ and cytotoxic αβ T cells expressing either CD8αα or CD8αβ. T cell activation was gated on CD3+CD41/61−CD8α+ cells expressing CD107 or on CD3+TCRγδ+CD8α+ and CD3+TCRγδ−CD8α+ cells expressing IFNγ, and only in spleen also CD3+TCRγδ−CD8α− (CD4+) cells expressing IFNγ. The marker CD41/61 is included in the CD107 assay to exclude thrombocytes from analysis, since activated thrombocytes have been reported to express CD107 [69]. In the CD107 assay NK cells are gated by excluding T cells and thrombocytes since a pan NK marker is missing while for phenotyping the NK cells are gated based on expression of the NK markers IL-2Rα and 20E5, which are known to be expressed on cells with NK function [48]. [file 13567_2022_1026_MOESM1_ESM.tif]

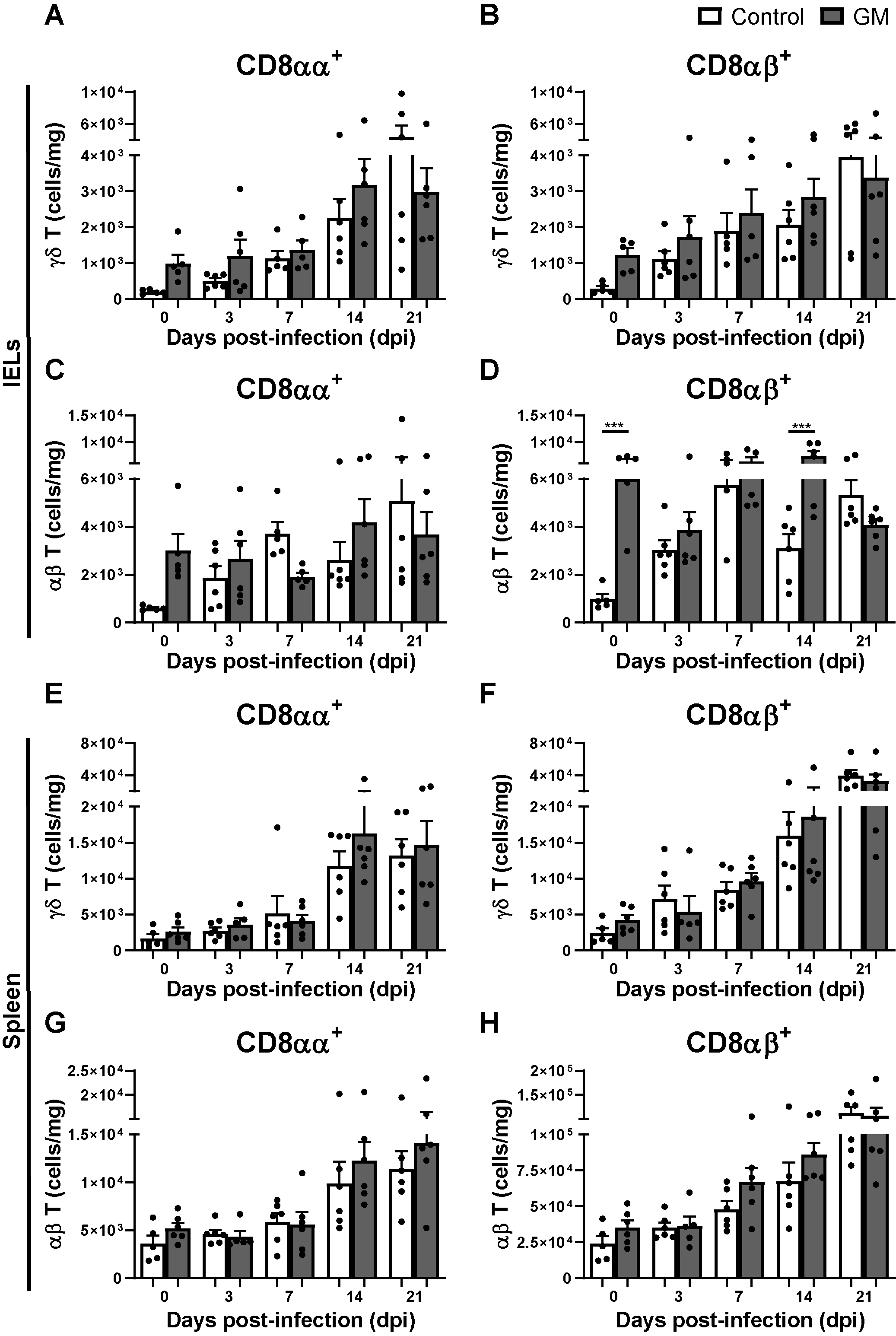

Supplement: Supplementary file 2 — Additional file 2. Effect of GM on numbers of intraepithelial and splenic γδ T cells and cytotoxic T cells expressing either CD8αα+ and CD8αβ+ before and during SE infection in broiler chickens. A Numbers (cells/mg) of intraepithelial CD8αα+ γδ T cells, B CD8αβ+ γδ T cells, C cytotoxic CD8αα+ T cells and D CD8αβ+ T cells in chickens either fed standard (control) or long-chain glucomannan supplemented (GM) diet in course of time before and during SE infection. E Numbers (cells/mg) of splenic CD8αα+ γδ T cells, F CD8αβ+ γδ T cells, G cytotoxic CD8αα+ T cells and H CD8αβ+ T cells in chickens either fed standard or GM diet before and during SE infection. Mean + SEM per diet group and time point are shown (n = 6), if n = 5; one chicken was excluded due to numbers of events acquired in the gate of interest were < 100. Statistical significance between diet groups is indicated as ***(p < 0.001). [file 13567_2022_1026_MOESM2_ESM.tif]

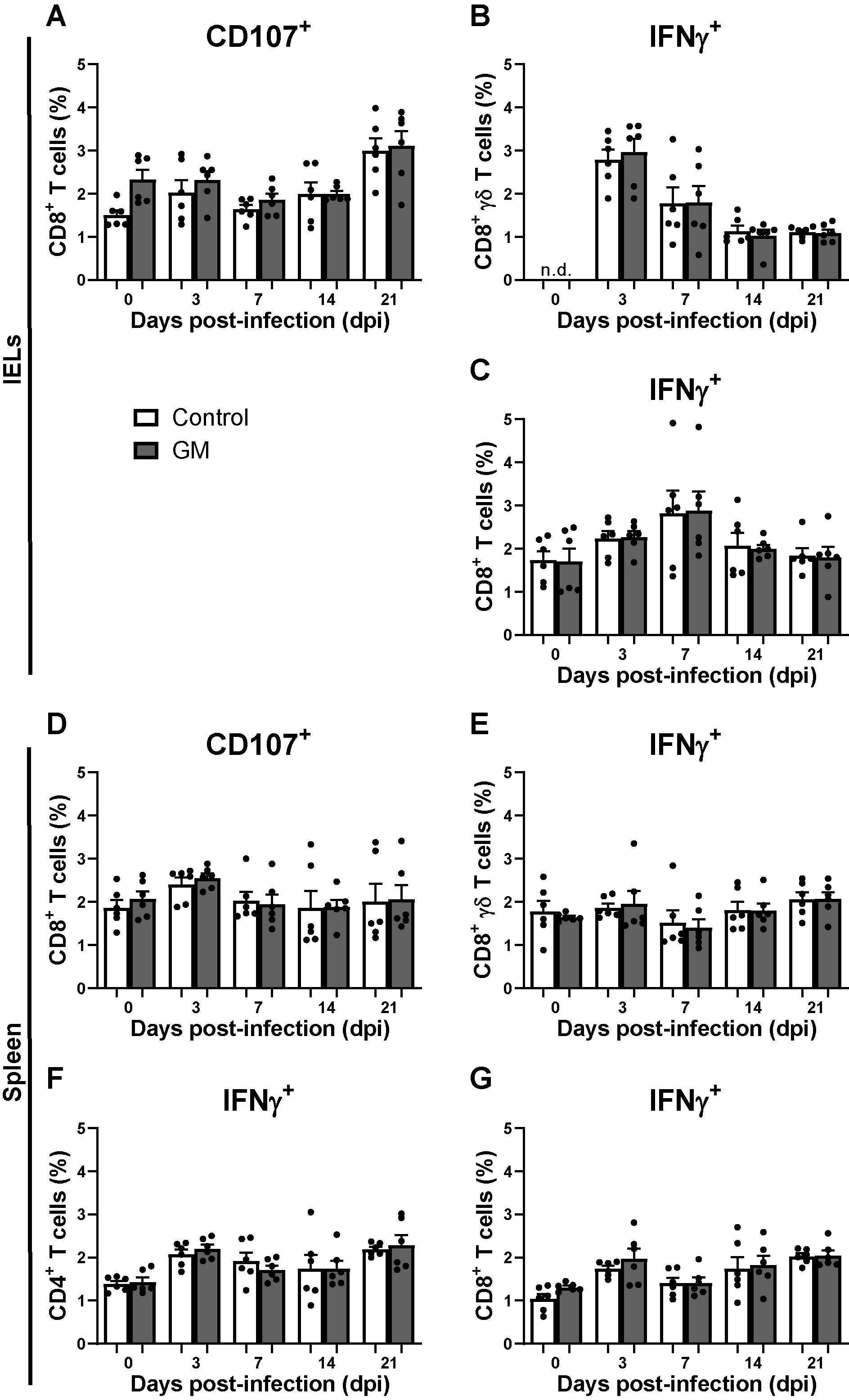

Supplement: Supplementary file 3 — Additional file 3. Effect of GM on T cell activation in IELs and spleen before and during SE infection in broiler chickens. A Percentages of intraepithelial CD8+ T cells expressing CD107 (including both γδ and αβ T cells), B CD8+ γδ T cells expressing IFNγ and C CD8+ αβ T cells expressing IFNγ in chickens either fed standard (control) or long-chain glucomannan supplemented (GM) diet in course of time before and during SE infection. D Percentages of splenic CD8+ T cells expressing CD107 (including both γδ and αβ T cells), E CD8+ γδ T cells expressing IFNγ, F CD4+ αβ T cells expressing IFNγ and G CD8+ αβ T cells expressing IFNγ in chickens either fed standard or GM diet before and during SE infection. Mean + SEM per diet group and time point are shown (n = 6), for IFNγ expression of CD8+ γδ T cells in the IEL population at 0 dpi percentages were not determined (nd) due to numbers of events acquired in the gate of interest were < 100. [file 13567_2022_1026_MOESM3_ESM.tif]

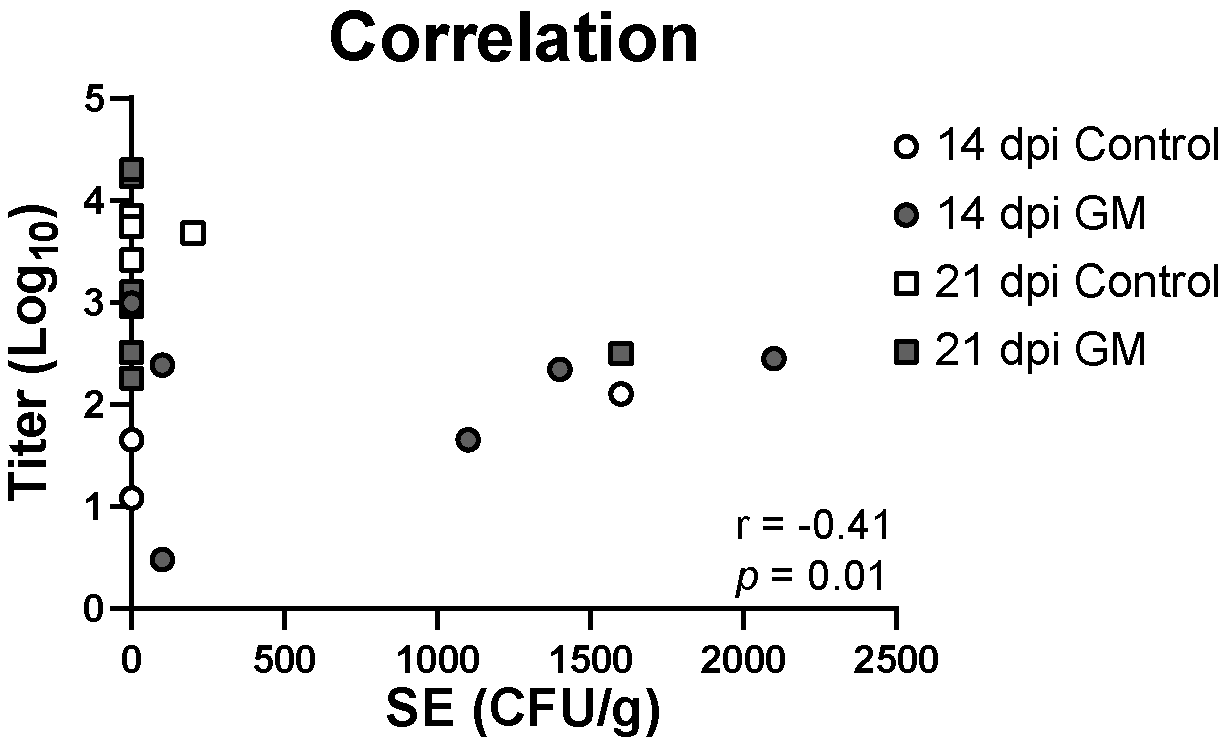

Supplement: Supplementary file 4 — Additional file 4. Correlation between serum antibody titers and SE-CFUs in broiler chickens. Correlation between SE-specific antibody titers and splenic SE-CFUs of chickens either fed standard (control) or long-chain glucomannan (GM) diet using the Spearman rank correlation test. Statistical significance is indicated as p = 0.01. [file 13567_2022_1026_MOESM4_ESM.tif]

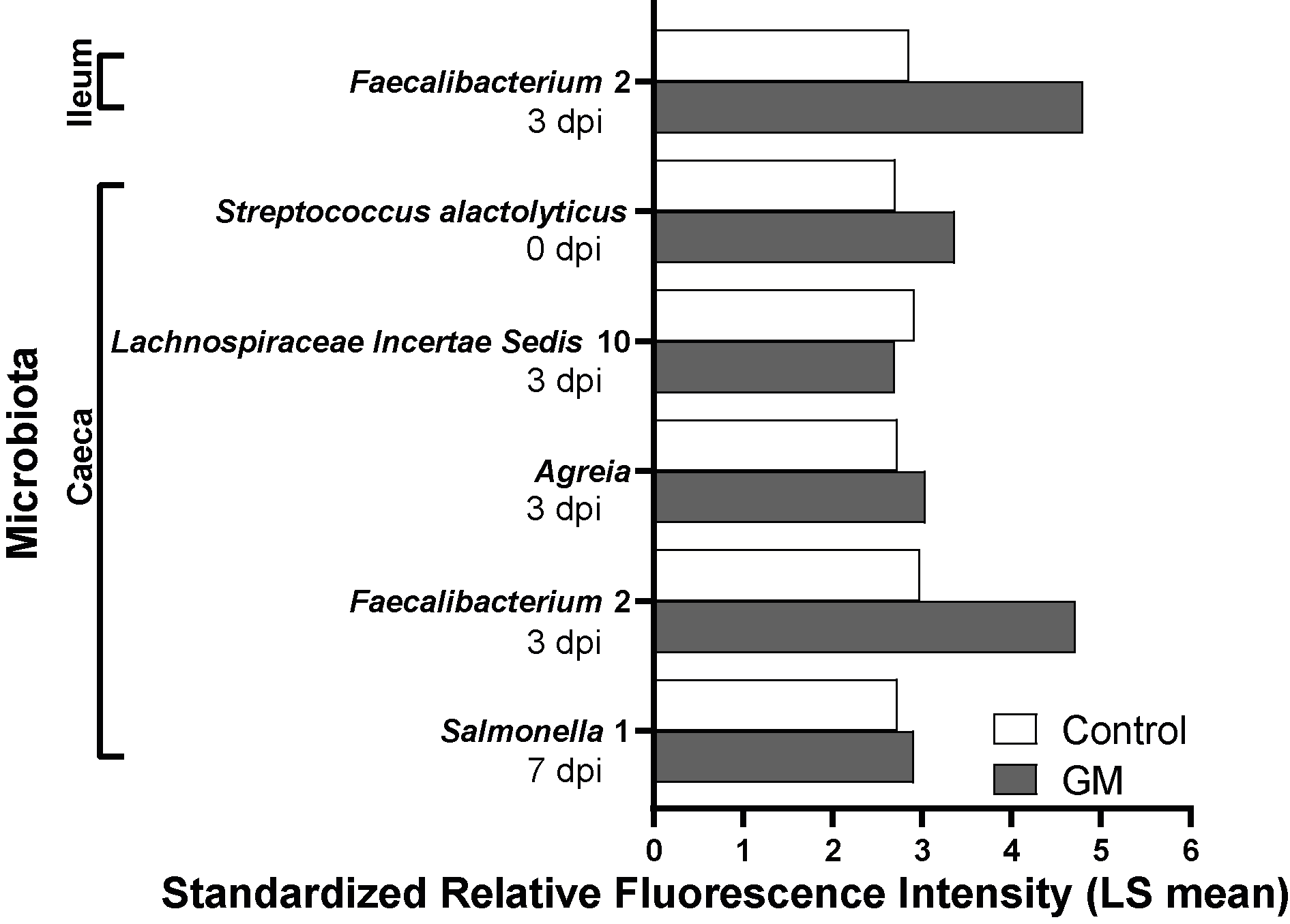

Supplement: Supplementary file 5 — Additional file 5. Intestinal microbial taxa significantly increased with diet at 0, 3 and 7 dpi of SE in broiler chickens. Standardized relative fluorescence intensities of the microbial taxa as measured by the microarray in the ileum and caeca (Table 3) that were significantly increased either with standard (control) or long-chain glucomannan supplemented (GM) diet at 0, 3 and 7 dpi of SE in broiler chickens. LS mean per microbial taxa and diet group are shown (n = 6) with statistical significance of FDR adjusted p values set at < 0.05. [file 13567_2022_1026_MOESM5_ESM.tif]

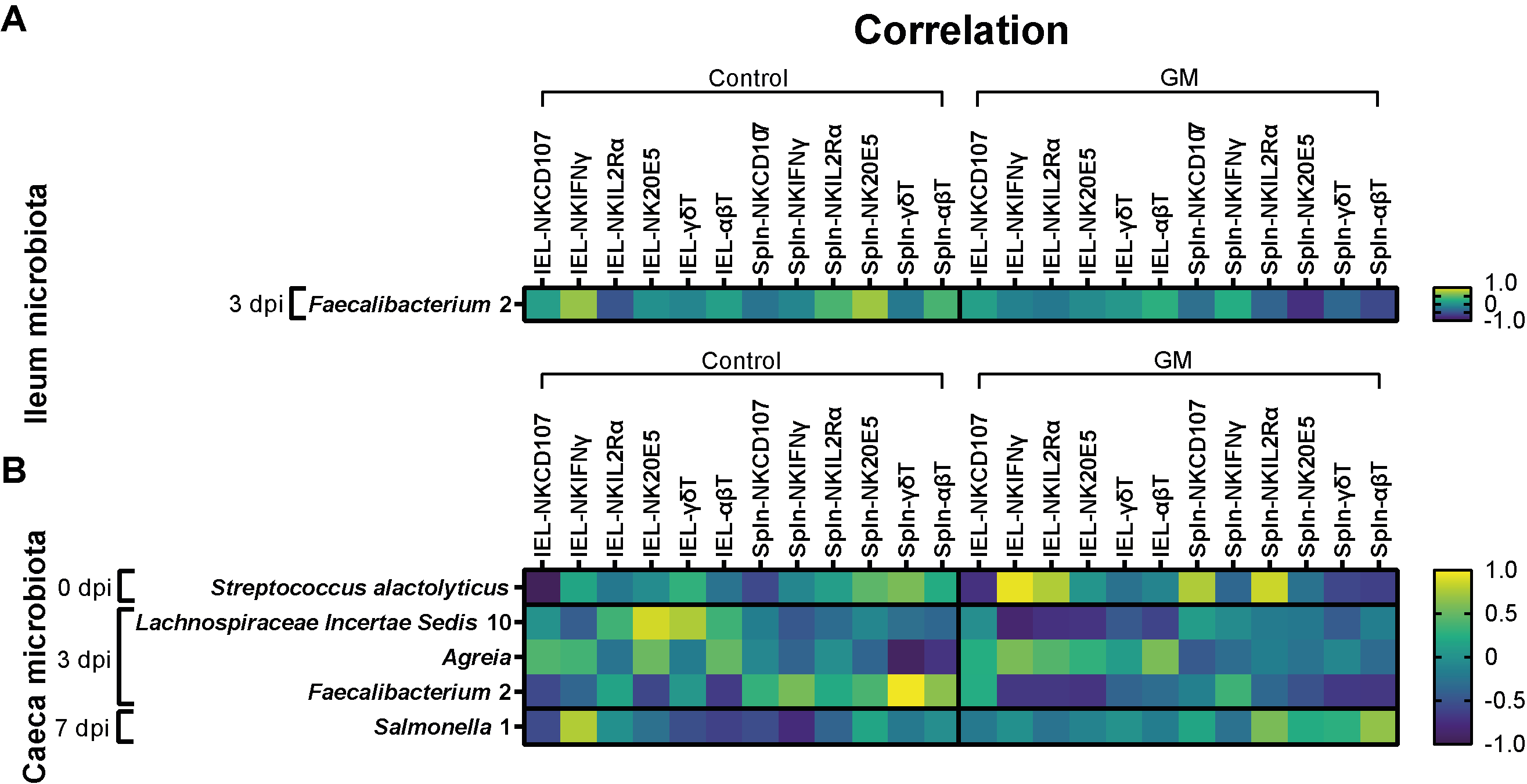

Supplement: Supplementary file 6 — Additional file 6. Correlation between microbial taxa and intraepithelial and splenic immune parameters at 0, 3 and 7 dpi of SE in broiler chickens. A Correlation values between intestinal microbial taxa in the ileum or B caeca significantly increased with diet and percentages of NK cell activation (CD107 or IFNγ expression) or numbers of NK and T cell subsets of the ileum (IEL) and spleen (Spln) per diet (control, GM) at 0, 3 and 7 dpi of SE in broiler chickens. Pearson’s correlation (r) values are depicted in a heatmap as positive (yellow) or negative (dark blue) correlations. [file 13567_2022_1026_MOESM6_ESM.tif]
